# Microorganisms Accelerate REE Mineralization in Supergene Environments

**DOI:** 10.1128/aem.00632-22

**Published:** 2022-06-16

**Authors:** Xurui Li, Xiaoliang Liang, Hongping He, Jintian Li, Lingya Ma, Wei Tan, Yin Zhong, Jianxi Zhu, Mei-Fu Zhou, Hailiang Dong

**Affiliations:** a CAS Key Laboratory of Mineralogy and Metallogeny/Guangdong Provincial Key Laboratory of Mineral Physics and Materials, Guangzhou Institute of Geochemistry, Chinese Academy of Sciences, Guangzhou, China; b CAS Center for Excellence in Deep Earth Science, Guangzhou, China; c University of Chinese Academy of Sciences, Beijing, China; d Institute of Ecological Science and Guangdong Provincial Key Laboratory of Biotechnology for Plant Development, School of Life Sciences, South China Normal University, Guangzhou, China; e Department of Earth Sciences, The University of Hong Kong, Hong Kong, China; f State Key Laboratory of Biogeology and Environmental Geology, China University of Geosciences, Beijing, China; g Department of Geology and Environmental Earth Science, Miami University, Ohio, USA; University of Michigan-Ann Arbor

**Keywords:** REE mineralization, microbial community, REE fractionation, biosorption, gene sequencing, Gram-positive bacteria, *Bacillus*, electron microscopy

## Abstract

Exogenic deposits are an important source of rare earth elements (REEs), especially heavy REEs (HREEs). It is generally accepted that microorganisms are able to dissolve minerals and mobilize elements in supergene environments. However, little is known about the roles of microorganisms in the formation of exogenic deposits such as regolith-hosted REE deposits that are of HREE enrichment and provide over 90% of global HREE demand. In this study, we characterized the microbial community composition and diversity along a complete weathering profile drilled from a regolith-hosted REE deposit in Southeastern China and report the striking contributions of microorganisms to the enrichment of REEs and fractionation between HREEs and light REEs (LREEs). Our results provide evidence that the variations in REE contents are correlated with microbial community along the profile. Both fungi and bacteria contributed to the accumulation of REEs, whereas bacteria played a key role in the fractionation between HREEs and LREEs. Taking advantage of bacteria strains isolated from the profile, Gram-positive bacteria affiliated with *Bacillus* and *Micrococcus* preferentially adsorbed HREEs, and teichoic acids in the cell wall served as the main sites for HREE adsorption, leading to an enrichment of HREEs in the deposit. The present study provides the first database of microbial community in regolith-hosted REE deposits. These findings not only elucidate the crucial contribution of fungi and bacteria in the supergene REE mineralization but also provide insights into efficient utilization of mineral resources via a biological pathway.

**IMPORTANCE** Understanding the role of microorganisms in the formation of regolith-hosted rare earth element (REE) deposits is beneficial for improving the metallogenic theory and deposit exploitation, given that such deposits absolutely exist in subtropical regions with strong microbial activities. Little is known of the microbial community composition and its contribution to REE mineralization in this kind of deposit. Using a combination of high-throughput sequencing, batch adsorption experiments, and spectroscopic characterization, the functional microorganisms contributing to REE enrichment and fractionation are disclosed. For bacteria, the surface carboxyl and phosphate groups are active sites for REE adsorption, while teichoic acids in the cell walls of G^+^ bacteria lead to REE fractionation. The above-mentioned findings not only unravel the importance of microorganisms in the formation of supergene REE deposits but also provide experimental evidence for the bioutilization of REE resources.

## INTRODUCTION

Rare earth elements (REEs), defined as a group of 17 elements including the 15 elements in the lanthanide group plus scandium (Sc) and yttrium (Y) (1), are indispensable to numerous cutting-edge technologies (2). Yttrium is commonly classified as a heavy REE (HREE) since it shares similar properties with other elements belonging to HREEs (3). Compared to light REEs (LREEs; La-Eu), heavy REEs (HREEs; Gd-Lu and Y) with a lower crustal abundance are extremely scarce (4). Along with the wide applications of HREEs in clean energy, aerospace, and defense industries, global demands for HREEs are increasing rapidly (3). In contrast to other metals that can be concentrated in endogenic deposits, HREEs are primarily associated with exogenic deposits such as placer deposits and regolith-hosted deposits (5). Other potential exogenic REE deposits include those associated with phosphorites, bauxites, coal seams, and deep-sea muds (6). To date, regolith-hosted REE deposits in granite terrains are a dominant source of HREEs and provide over 90% of global HREE products (7).

Exogenic deposits are commonly formed through the secondary enrichment of REEs in supergene environments via decomposition and/or transportation of REE-bearing minerals. Weathering-susceptible REE-bearing minerals (e.g., REE fluorocarbonates, allanite, titanite, and apatite) can be readily weathered and decomposed such that REE cations are mobilized (8). Dissolved REEs can be adsorbed onto clay minerals and Fe/Mn (oxyhydr)oxides to form supergene REE deposits in regolith (9). Refractory REE-bearing minerals (e.g., monazite, xenotime, and fergusonite) can survive weathering processes and may form residual placers or eluvial and beach placers (10). Additionally, biogenic apatite may scavenge REEs through ion-exchange or structural substitution, giving rise to the formation of REE resources in the form of phosphorite, coal seam, and deep-sea mud (6).

Regolith-hosted REE deposits are also known as ion adsorption-type REE deposits as well as weathered crust elution-deposits. The presence of easily weathered HREE minerals [e.g., synchysite-(Y), gadolinite, and titanite] in the granitic bedrock is the primary control of HREE-rich deposits (7). The dissolved HREEs form stronger complexes with humic matter and carbonate ions relative to LREEs, promoting the downward migration of HREEs (11, 12), which makes HREEs available for adsorption. With an increase of soil pH during the downward transport of REEs, they are immobilized by secondary minerals through adsorption or incorporation. Clay minerals including kaolinite and halloysite are the dominant adsorbents for ion-exchangeable REEs, without giving rise to REE fractionation (13, 14), whereas Fe-Mn (hydr)oxides preferentially scavenge HREEs (15, 16). The coupling of the above-described processes on the mobility and fractionation of REEs manifests the complicated mechanisms underlying the formation of regolith-hosted REE deposits (17).

Although physical and chemical processes involved in the formation of regolith-hosted REE deposits have been well documented, little is known about the role of microbes in such deposits. Exogenic deposits formed in supergene environments with microbial activities. It has been reported that 1 g of soil can contain up to 10 billion microorganisms with thousands of different species (18, 19). The ubiquitous microorganisms in supergene environments can trigger intense microbe-mineral interactions (20). Alteration of minerals and growth of microorganisms are coupled processes in nature. Minerals and rocks provide microorganisms with nutrients and living habitats, whereas microorganisms affect the weathering and diagenesis of rocks and minerals through dissolution, transformation, and formation of secondary minerals (21, 22). Several microbial species, such as model bacteria Bacillus subtilis (23) and wild-type Variovorax paradoxus (24), have been found capable of adsorbing REEs. Some of those even possess highly selective adsorption capacities through complexation with specific functional groups on cell surface, leading to fractionation between LREEs and HREEs (25). Thus, microorganisms may play a vital role in the enrichment of specific REEs in supergene environments (26). Previous studies have focused mainly on microbial communities in soils that are not associated with REE deposits (27, 28). Systematic investigations of microbial distribution and correlation with REE variations across an entire weathering profile within REE deposits are still lacking but are of high importance for understanding the roles of microorganisms in REE mineralization in supergene environments.

We used samples collected from a weathering profile of the Renju REE deposit, a well-known regolith-hosted deposit in Southeastern China, to examine the controls of microorganisms on REE mineralization in supergene environments (Fig. S1). We employed a high-throughput sequencing method to characterize the microbial community composition along a 45-m-thick regolith profile sampled through a drillhole, which was linked to the distribution and fractionation of REEs in the profile. To reveal the REE enrichment and fractionation mechanisms by microbes, laboratory adsorption experiments were also carried out using a variety of bacteria isolated from the profile. We demonstrate that both fungi and bacteria can contribute to the REE mineralization in regolith-hosted deposits and the selective adsorption of REEs by teichoic acids in the cell walls of Gram-positive bacteria can contribute to the fractionation between HREEs and LREEs.

## RESULTS

### Variation of mineralogy along the weathering profile.

**FIG 1:**
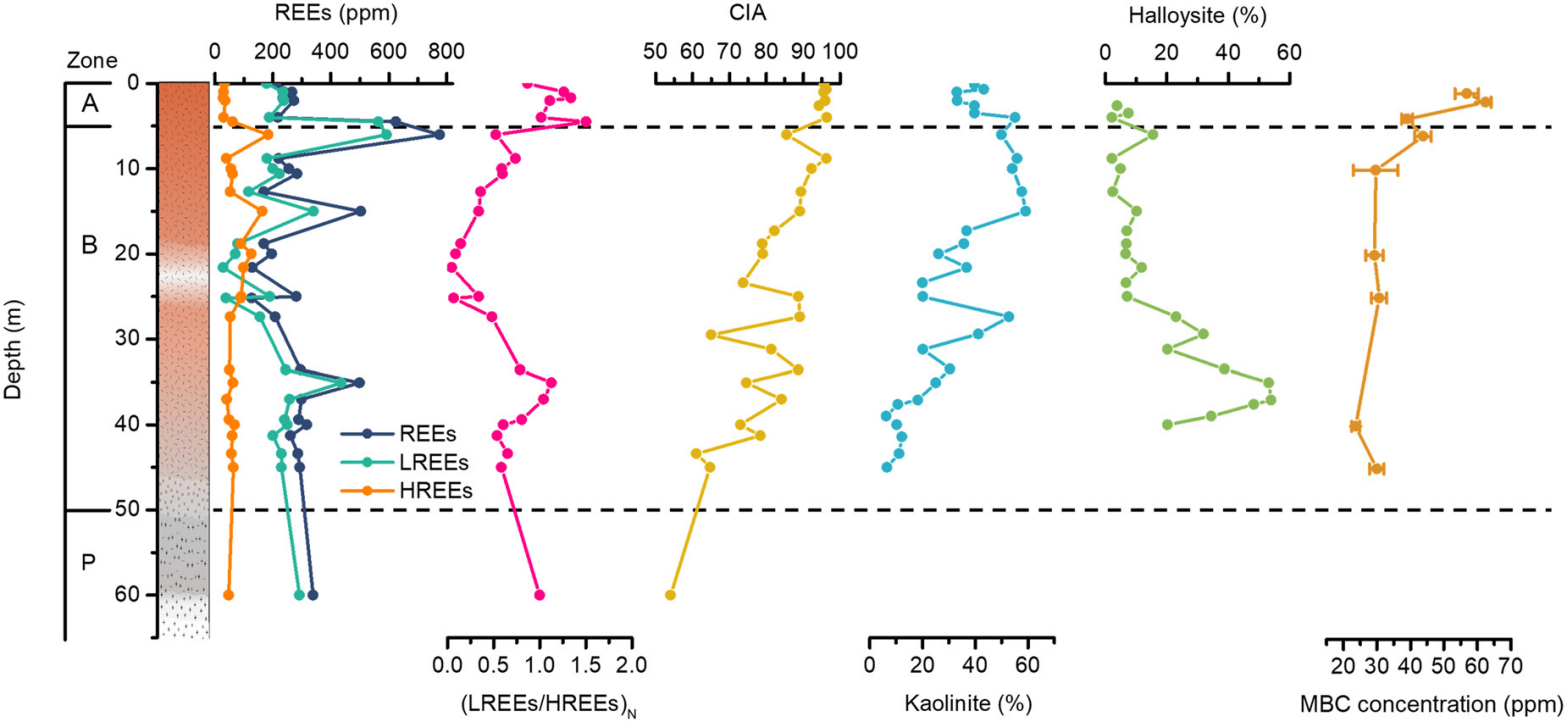
Depth variations in the content of rare earth elements (REEs), chemical index of alteration (CIA), mineral abundance, and microbial biomass carbon (MBC) concentration. The total content of REEs and (LREEs/HREEs)_N_ ratio are considered an indicator of REE enrichment and fractionation, respectively. (LREEs/HREEs)_N_ is the ratio of LREEs/HREEs in the weathering profile to that in the bedrock. (LREEs/HREEs)_N_ > 1 and (LREEs/HREEs)_N_ < 1 indicate the relative enrichment of LREEs and HREEs, respectively, in the weathering profile compared to bedrock.

According to the texture, color, and mineral composition (See Text S1 for description), the whole weathering profile of the Renju deposit can be divided into three zones with different chemical indexes of alteration (CIA): A horizon of the topsoil and complete weathering layer with the highest CIA (CIA > 95), B horizon dominated by the incompletely weathered layer (CIA = 60 to 95), and P horizon composed of the weathering front and bedrock (CIA < 60). The thickness of the A and B horizons ranges from 0 to ~5 m and ~5 to ~50 m, respectively (Fig. 1).

Variations in the contents of minerals along the weathering profile are shown in Table S1. The P horizon is fine-grained (0.5 to 2.0 mm) equigranular quartz diorite consisting of plagioclase (ca. 53%), amphibole (ca. 14%), biotite (ca. 10%), chlorite (the hydrothermally altered products of amphibole and biotite; ca. 10%), quartz (ca. 10%), and K-feldspar (ca. 4%). The REE-bearing minerals include REE fluorocarbonates, titanite, apatite, allanite, monazite, xenotime, and zircon. Owing to the different weathering resistance (29), REE fluorocarbonates, titanite, apatite, and allanite exist only in the P and lower B horizons, while monazite, xenotime, and zircon are preserved in the A or upper B horizon (Fig. S2).

From the bedrock to the A horizon, the quartz content gradually increases from ca. 10% to more than 60%, while plagioclase gradually disappears. The presence of K-feldspar is constant at ca. 4% along the weathering profile, with two exceptionally high contents of ca. 13% and ca. 19% occurring at the bottom (~45 m) and the middle (~20 m) of the B horizon, respectively.

As for the weathering-susceptible minerals such as amphibole, chlorite, and biotite, they disappear at the bottom of the B horizon (~45 m), accompanied by the occurrence of illite (ca. 29%) and smectite (ca. 20%) (Fig. S3 and Table S1). With the progress of weathering, the clay minerals in the B horizon gradually vary from smectite and illite to kaolinite and halloysite. Kaolinite appears at ~45 m with a content of 6%, then gradually becomes predominant to the top of the B horizon and the bottom of the A horizon (50 to 55%, 4 to 10 m), and decreases in content to 33% at the surface of the profile (1 to 2 m). As to halloysite, its content gradually decreases from 20% at ~40 m to 2% at ~4 m, with an exceptional second highest content (15%) present at ~6 m (Fig. 1). The A horizon is composed mainly of kaolinite and some rounded residual quartz grains, with a small amount of hematite and gibbsite.

### REE contents across the weathering profile.

In both the A and B horizons of the weathering profile in the Renju deposit, the contents of total REEs and LREEs are generally lower than those of the bedrock (Fig. 1, Table S2). The variation of REE content is small (195 to 317 ppm). Nearly all the detected REE contents are lower than the average value of bedrock (339 ppm), except for an abrupt increase to a maximum of 776 ppm at the top of B horizon (ca. 6 m). The distribution of LREEs is identical to that of total REEs. Most LREE contents range from 70 to 250 ppm, lower than that of bedrock (292 ppm), while the maximum is 592 ppm at ca. 6 m. As for HREE contents, though the location of the maximum (184 ppm) overlaps with those of total REEs and LREEs at ca. 6 m, the variations with depth are obviously different. Compared to the bedrock with an average HREE content of 47 ppm, the A horizon is poor in HREEs (<35 ppm), but the B horizon is rich in HREEs (56 to 184 ppm), with two high concentrations (184 and 125 ppm) at ca. 6 m and ca. 15 m of the upper B horizon, respectively.

The different distributions of the total LREEs and HREEs give rise to a significant fluctuation of LREEs/HREEs ratio along the profile. Despite the fact that the bedrock displays a remarkable depletion of HREEs with an average LREEs/HREEs ratio of 6.1, the ratio of LREEs/HREEs in the weathering profile relative to that in the bedrock [labeled (LREEs/HREEs)_N_] gradually decreases to 0.05 at the middle part of the B horizon (ca. 21.6 m) from the lower part but then increases to 1.5 near the surface of the profile (ca. 4.5 m). This indicates the relative enrichment of LREEs and HREEs in the A and B horizons, respectively. With the variation of weathering degree, the pH increases from 4.3 at the topsoil to 7.0 at the bottom of B horizon.

### Microbial biomass, diversity, and community composition along the weathering profile.

Microbial biomass carbon (MBC) was measured by chloroform fumigation extraction method (30). Along the weathering profile, MBC concentrations are high in the A horizon and top of B horizon (ca. 6 m) but low in the lower part of B horizon (Fig. 1). Specifically, the highest MBC concentration (62.5 ppm) is located at ca. 2 m and the lowest (23.6 ppm) at ca. 40 m, respectively (Table S3).

To measure the microbial α-diversity, Chao1 and Shannon indices were calculated. Bacterial and fungal diversities do not decline systematically with increasing depth (Fig. S5). Bacterial diversity shows a roughly U-shaped pattern. For both Chao1 and Shannon indices, the upper and lower parts of the profile have the most bacterial diversity. However, fungal diversity is much lower than that of bacteria and has roughly a W-shape along the profile. Based on Chao1 index, three peaks of fungal diversity appear at the top of A horizon (ca. 1 m) and the top (ca.10 to 20 m) and bottom (ca. 45 m) of B horizon. Among them, the diversity at the bottom section is the highest. When Shannon index is taken into account, the peaks of fungal diversity overlap those of Chao 1 index at the top of A horizon and the bottom of B horizon, except for the top of B horizon (ca. 6 m) harboring the maximum fungal diversity.

**FIG 2:**
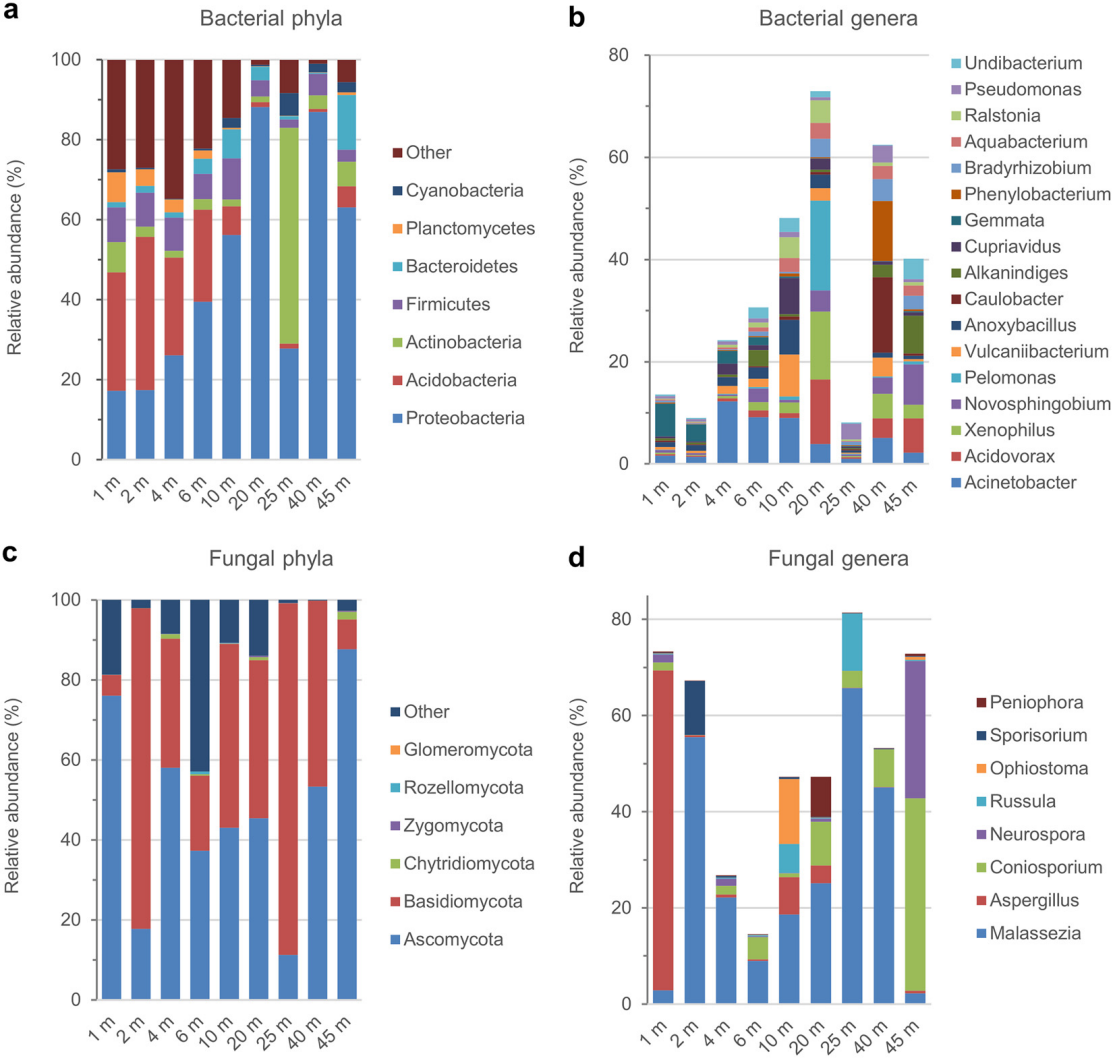
Composition of microbial communities in the Renju deposit profile. (a) Relative abundances of dominant bacterial phyla (with an average relative abundance of >1% across the profile). The “other” group represents those with an average relative abundance of <1%. (b) Relative abundances of dominant bacterial genera (>1%). (c) Relative abundances of fungal phyla. (d) Relative abundances of dominant fungal genera (>1%).

Considering that analyses of microbial community composition in the literature were generally focused on phyla and genera (27, 31), these two taxonomic levels were distinguished in our data analysis. In total, 37 different bacterial phyla were detected across the deposit profile. Among them, *Proteobacteria*, *Acidobacteria*, *Actinobacteria*, *Firmicutes*, *Bacteroidetes*, *Planctomycetes*, and *Cyanobacteria* are numerically dominant, with relative abundances of >1% individually (Fig. 2a, Table S4). These seven dominant phyla are present along the whole profile and together account for 84.2% of the total 16S rRNA gene sequences. Unexpectedly, the relative abundance of *Proteobacteria* (i.e., the first abundant bacterial phylum, with an average relative abundance of 46.9% across all the samples) rises with increasing depth to the middle of B horizon (ca. 20 m) but then decreases more or less in the lower profile. In contrast, the relative abundance of *Acidobacteria* (i.e., the second most abundant bacterial phylum, 14.6%) tends to decrease with increasing depth to the lower part of B horizon (ca. 40 m) before a slight increase at the bottom (ca. 45 m). *Actinobacteria* (the third abundant phylum, 9.01%) exhibits an abnormally high relative abundance at the middle of B horizon (ca. 25 m, 54.0%), while its relative abundances in other sampling depths are relatively low.

A total of 700 bacterial genera were detected across the profile. The dominant genera (i.e., those with an average relative abundance of >1%) are *Acinetobacter*, *Acidovorax*, *Xenophilus*, *Novosphingobium*, *Pelomonas*, *Vulcaniibacterium*, *Anoxybacillus*, *Caulobacter*, *Alkanindiges*, *Cupriavidus*, *Gemmata*, *Phenylobacterium*, *Bradyrhizobium*, *Aquabacterium*, *Ralstonia*, *Pseudomonas*, and *Undibacterium* (Fig. 2b). They are widely distributed along the profile, together accounting for 41.4% of the total 16S rRNA gene sequences. The contribution of these dominant genera to the total bacterial community increases with depth to the middle of profile (ca. 20 m) but then decreases more or less in the lower profile. *Acinetobacter* is the most abundant genus with the highest relative abundance of 12.2% at ca. 4 m.

As for fungi, a total of six known phyla were detected (Fig. 2c). The dominant phyla are *Ascomycota* and *Basidiomycota*, whose abundances are 47.8% and 40.4% on average along the profile, respectively, together accounting for more than 88% of the total internal transcribed spacer (ITS) sequences. *Malassezia*, *Aspergillus*, *Coniosporium*, *Neurospora*, *Russula*, *Ophiostoma*, *Sporisorium*, and *Peniophora* were identified as the dominant fungal genera across the profile (Fig. 2d). *Aspergillus* and *Coniosporium* are the first dominant genera at the top of A horizon (~1 m) and the bottom of B horizon (~45 m), respectively. The fungal communities at other sampling depths are dominated by *Malassezia*.

### Correlations between REE and microbial taxa distribution.

**FIG 3:**
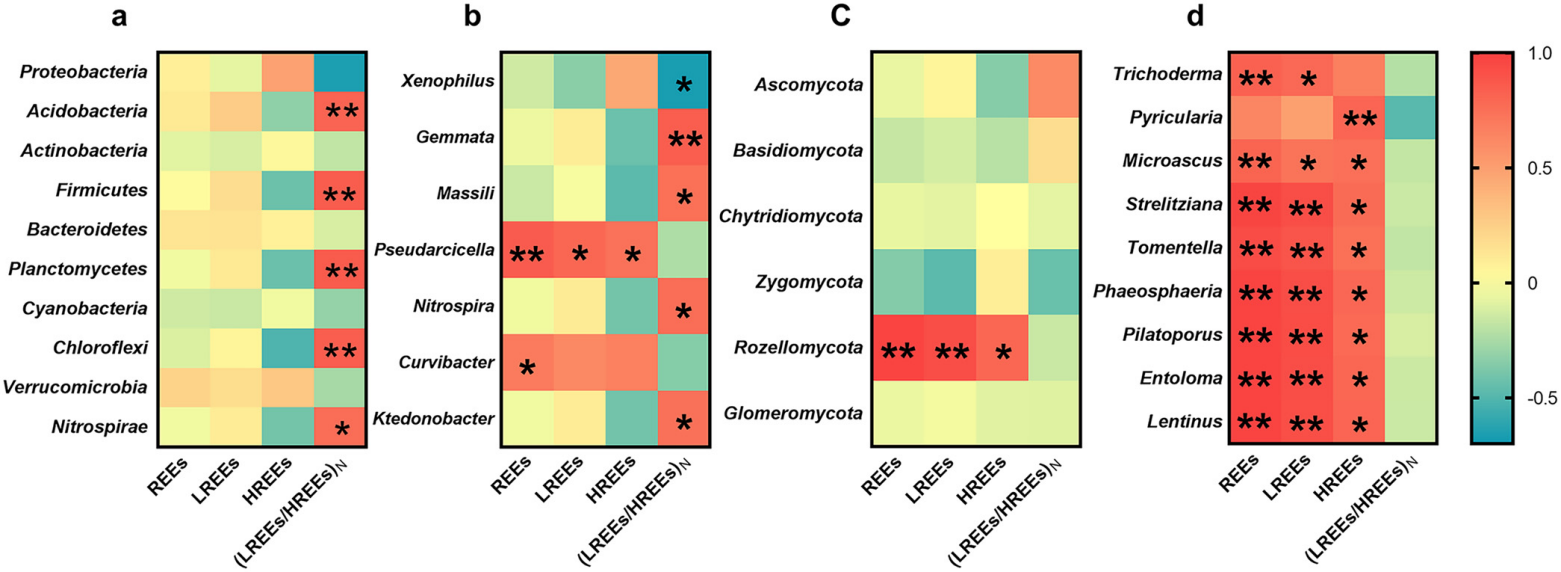
Correlations between REEs and concentrations of dominant microbial taxa in the Renju deposit profile. (a) Relationships between REEs and the concentrations of the top 10 dominant bacterial phyla. (b) Relationship between REEs and the concentrations of abundant bacterial genera (>0.1%). (c) Relationships between REEs and the concentrations of fungal phyla. (d) Relationships between REEs and the concentrations of abundant fungal genera (>0.1%). Only those genera with significant correlations with REEs are shown in panels b and d. *, *P* < 0.05; **, *P* < 0.01. Color bar scale represents correlation coefficient. *r* > 0, positive correlation; *r* < 0, negative correlation.

The associations between REE enrichment/fractionation and microbial taxa concentrations are revealed by Pearson correlation analysis (Fig. 3, Table S5). Here, the (LREEs/HREEs)_N_ ratio and total content of REEs (including the scenario that LREEs and HREEs are taken into account separately) are considered an indicator of REE fractionation and enrichment, respectively. Concentration of each dominant microbial taxon is calculated by multiplying their relative abundance by the total MBC concentration of soil samples collected at different depths. Note that the calculation of the concentrations of dominant microbial taxa is a compromise solution, due to the lack of suitable methods at present. Among the top 10 dominant bacterial phyla, *Acidobacteria*, *Firmicutes*, *Planctomycetes*, *Chloroflexi*, and *Nitrospirae* are found to have significant positive correlations with REE fractionation (all *r* > 0.76, *P < *0.05; Fig. 3a). When abundant bacterial genera (with an average relative abundance > 0.1% across the profile) are taken into account, the distributions of *Gemmata* (belonging to *Planctomycetes*), *Massilia* (*Proteobacteria*), *Nitrospira* (*Nitrospirae*), and *Ktedonobacter* (*Chloroflexi*) have positive correlations with the (LREEs/HREEs)_N_ ratios (all *r* ≥ 0.74, *P < *0.05; Fig. 3b). Among the dominant bacterial genera, only *Xenophilus*, belonging to *Proteobacteria*, shows a negative correlation with (LREEs/HREEs)_N_ (*r* = −0.67, *P < *0.05). However, this is largely due to a scenario that *Xenophilus* has a tremendous relative abundance of 13.3% at 20 m. When the extreme data point is excluded, there is no significant correlation between *Xenophilus* and (LREEs/HREEs)_N_. Additionally, *Pseudarcicella*, belonging to *Bacteroidetes*, a Gram-negative phylum, is significantly positively correlated with total content of REEs, LREEs, and HREEs. *Curvibacter*, belonging to *Proteobacteria*, is positively correlated with total content of REEs.

However, in the six fungal phyla, only *Rozellomycota* is related to REE enrichment (all *r* > 0.79, *P* < 0.05; Fig. 3c). Nine abundant fungal genera (>0.1%), including *Trichoderma*, *Pyricularia*, *Microascus*, *Strelitziana*, and *Phaeosphaeria* belonging to *Ascomycota* and *Tomentella*, *Pilatoporus*, *Entoloma*, and *Lentinus* belonging to *Basidiomycota*, are also positively correlated with the REE content (all *r* > 0.74, *P* < 0.05; Fig. 3d). However, they exhibit no significant relationships with the (LREEs/HREEs)_N_ ratio.

The correlations between MBC and REE fractionation/enrichment were calculated. There is a significant positive correlation between MBC and (LREEs/HREEs)_N_ ratio (*r* = 0.76, *P* < 0.05), while no significant relationship exists between MBC and REE enrichment (detailed data not shown).

### REE enrichment and fractionation by bacteria.

To unravel the contribution of bacteria to enrichment and fractionation of REEs, adsorption experiments were carried out by using nine bacteria strains of different genera that were isolated from the Renju deposit profile. The studied bacteria included two Gram-positive (G^+^) bacteria, i.e., Bacillus pumilus belonging to *Firmicutes* and *Micrococcus* sp. belonging to *Actinobacteria*, and seven Gram-negative (G^−^) bacteria, i.e., Pandoraea sputorum, Dyella thiooxydans, Herbaspirillum frisingense, Sphingomonas aquatilis, *Porphyrobacter* sp., Acidovorax caeni, and Delftia acidovorans belonging to *Proteobacteria*. Among them, *Acidovorax* is the second most dominant genus in the profile, with an average relative abundance of 3.00%, and *Sphingomonas* has an average relative abundance of 0.53%. In addition, the relative abundances of *Bacillus* and *Micrococcus* are approximately 0.05%. Other isolated genera have relatively low relative abundances.

**FIG 4:**
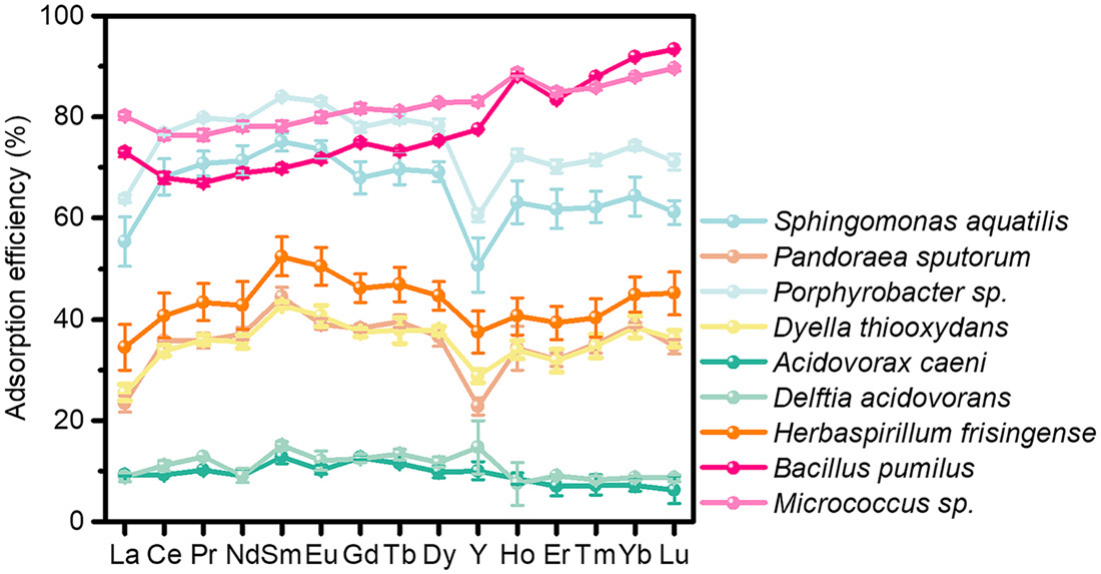
Adsorption of REEs by different bacteria isolated from the Renju deposit. pH = 5.0, temperature = 25°C, equilibrium time = 60 min; the initial concentration of each REE was 1 ppm.

After 1 h of adsorption of complete REE series on bacteria at an almost invariable pH of 5.0, these bacteria displayed variable affinities toward REEs, with an increasing order of adsorption efficiency: A. caeni (9.4%), D. acidovorans (10.9%), P. sputorum (35.2%), D. thiooxydans (35.4%), H. frisingense (43.3%), S. aquatilis (65.6%), *Porphyrobacter* sp. (74.9%), B. pumilus (77.6%), and *Micrococcus* sp. (82.4%) (Fig. 4). Most bacteria scavenged considerable REEs, except for *Acidovorax* and *Delftia*. In addition to remarkable enrichment, some bacteria also led to REE fractionation. Herein, Gram-positive bacteria (G^+^; i.e., *Bacillus* and *Micrococcus*) had the highest REE adsorption ability and showed a preferential adsorption toward HREEs (82.9% and 85.1%) relative to that toward LREEs (69.8% and 78.2%). The REE distribution patterns were leftward-inclined curves, characteristic of increasing adsorption with atomic number. In contrast, though the adsorption efficiency differed from strain to strain, G^−^ bacteria (the other seven bacteria) did not show any adsorption selectivity between HREEs and LREEs, with relatively flat patterns for the adsorbed REEs. Only the tetrad effect and Y-negative anomaly enhanced with the increase of adsorption efficiency.

### REE adsorption characterization by representative bacteria.

**FIG 5:**
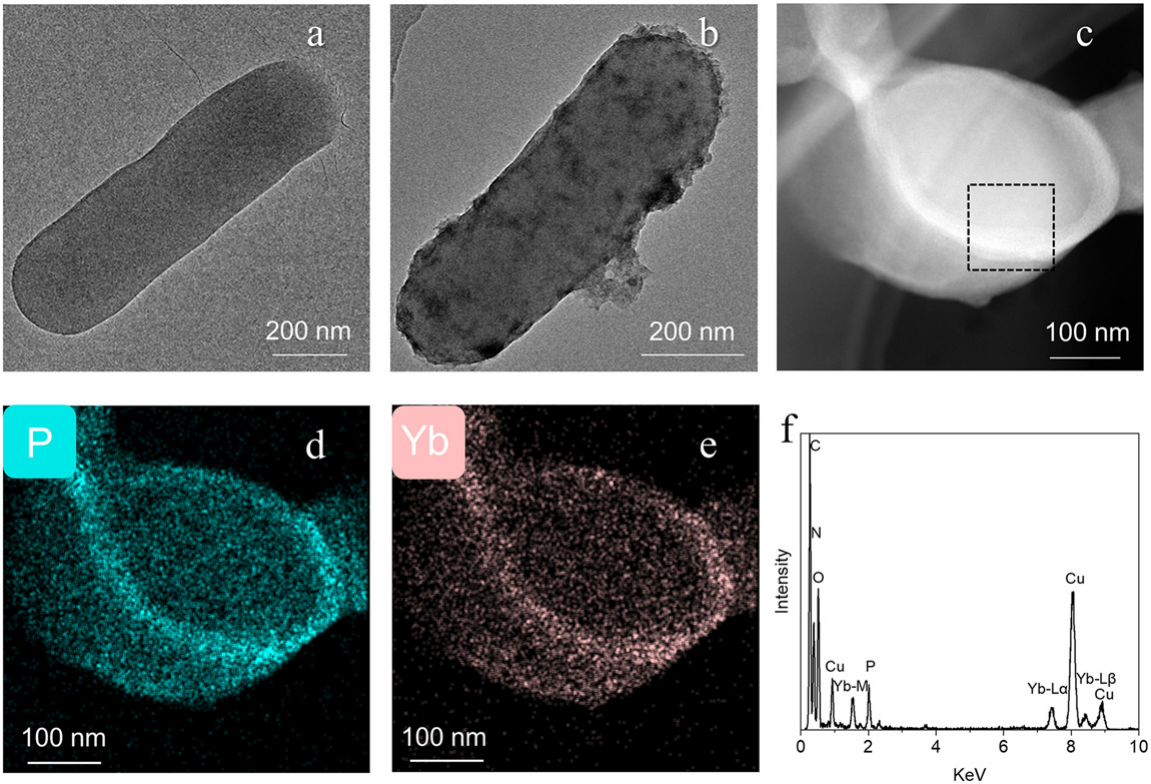
Distribution and adsorption species of ytterbium [Yb(III)] in the cells. (a and b) TEM images of *B. pumilus* before and after adsorption of Yb(III). (c) High-angle annular dark-field scanning transmission electron microscopy (HAADF-STEM) image of the cross-section of *B. pumilus* after adsorption of Yb(III). (d and e) The corresponding elemental mappings of P and Yb of panel c. (f) EDS analysis of the marked area in panel c showed that Yb(III) adsorbed by bacteria is bonded to the phosphate group on cell surface. The peaks of Yb-Lα, Yb-Lβ1, and Yb-Mα1 appear at 7.4, 8.4, and 1.5 KeV, respectively. The Yb-Lβ2 peak (8.8 KeV) overlaps with that of Cu-Kβ (8.9 KeV), due to the presence of Cu in the holder.

The adsorption sites of REEs on bacteria were further probed, which helps to understand the adsorption mechanism and the resulting fractionation effect. Herein, *B. pumilus* was selected as a representative G^+^ bacterium. *B. pumilus* suspension was mixed with Yb(III) solution for 2 h and then observed by transmission electron microscopy (TEM) and elemental mapping. The Yb(III) concentration was increased to 20 ppm, which enhanced the signal on element distribution. Compared to the control *B. pumilus* with a smooth cell surface, adsorption of Yb(III) generates floccule coating the bacterial surface (Fig. 5a and b). Ultrathin sections were sliced to reveal the element distributions in the cells (Fig. 5c). Unlike C, N, and O with an even distribution within bacterial cell, Yb is colocalized with P, where both elements are relatively rich along the cell wall (Fig. 5d to e, Fig. S8 and S10). Energy-dispersive X-ray spectrometer (EDS) analysis showed that Yb(III) adsorbed by bacteria is bonded to the phosphate group on cell surface (Fig. 5f). The close spatial association of Yb with P suggests that phosphate group on bacterial surface is the main site for Yb(III) adsorption (32).

**FIG 6:**
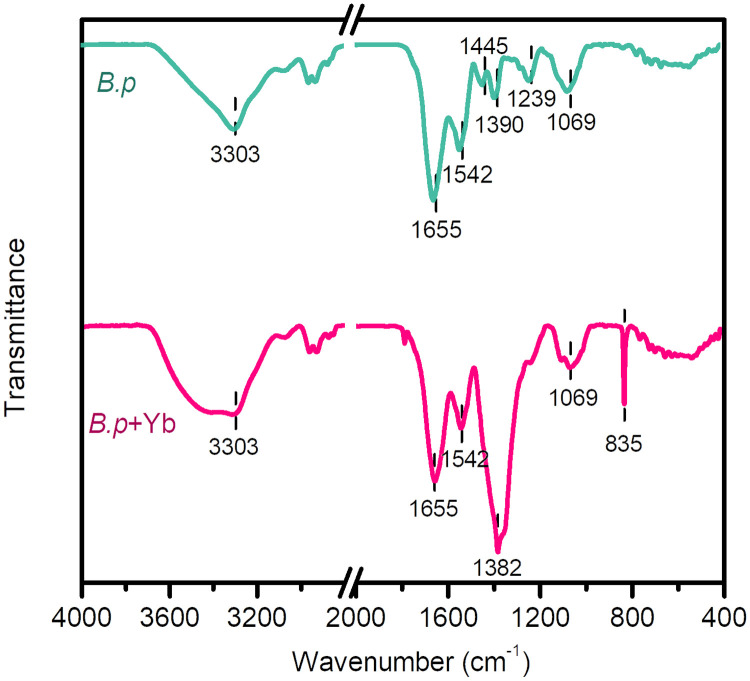
FTIR spectra of *B. pumilus* before and after the adsorption of Yb(III).

The change of surface groups on cell surface also certifies the adsorption sites for REEs, which was reflected by Fourier-transform infrared (FTIR) analyses (Fig. 6). For *B. pumilus*, the spectrum is divided into two regions, i.e., 2,800 to 3,700 and 700 to 2,000 cm^−1^. The former region displays a broad band at 3,000 to 3,700 cm^−1^ assigned to the stretching vibration of N-H (NH_2_^+^) and O-H (33), with several bands at 2,800 to 3,000 cm^−1^ affiliated with the asymmetric and symmetric stretching vibrations of C-H bond (34). In the region of 700 to 2,000 cm^−1^, the two strong bands at 1,655 and 1,542 cm^−1^ correspond to the C=O stretching vibration in *Amide I* and C-N-H bending modes in *Amide II*, respectively (35). The weak bands at 1,445, 1,390, 1,239, and 1,069 cm^−1^ are attributed to the deformation of C-H, C=O symmetric stretch of COO−, and *P*=O and P-O-P vibrations in phosphoester (36), respectively. The infrared (IR) spectrum of *B. pumilus* with adsorbed Yb(III) displays notable variations in the region of 700 to 2,000 cm^−1^. The sharp and strong bands at 1,382 and 835 cm^−1^ should be attributed to the newly formed complexes of Yb(III)-carboxyl (34) and Yb(III)-phosphate groups (37), probably corresponding to the breakdown of the protein structure of *B. pumilus* due to the complexation of Yb(III) with carboxyl group. This suggests that the carboxyl and phosphate groups of bacteria are the main adsorption sites for REEs, consistent with previous studies (34, 38, 39).

**FIG 7:**
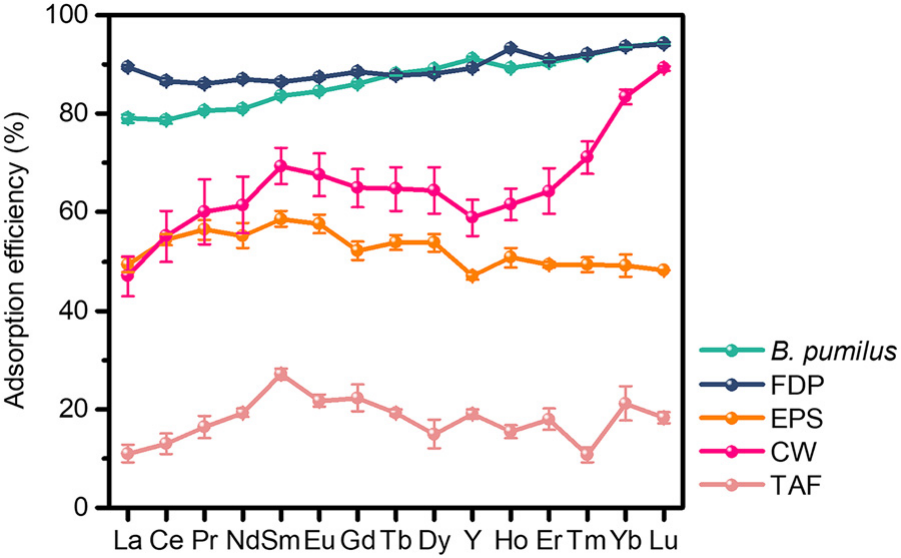
Adsorption of REEs on different bacterial structural components of *B. pumilus*. FDP, freeze-dried powder; EPS, extracellular polymeric substances; CW, cell wall; TAF, teichoic acid-free CW. Error bars indicate standard deviations from the means of the replicates.

Since all the investigated bacteria have carboxyl and phosphate groups on their surface, our finding of the contribution of bacteria to REE mineralization cannot well explain the preferential adsorption of HREEs by G^+^ bacteria, i.e., *Micrococcus* sp. of *Actinobacteria* and Bacillus pumilus of *Firmicutes*. Here, to understand the mechanism for the preferential adsorption of G^+^ toward HREEs, we further investigated the adsorption characteristics of REEs onto the control *B. pumilus*, its freeze-dried powder (FDP), and different cellular components, including extracellular polymeric substances (EPS), cell wall (CW), and teichoic acid-free CW (TAF) (Fig. 7). For control *B. pumilus*, the adsorption efficiency toward REEs achieved 86.8% at pH 5.0 within 1 h, with the capture of more HREEs (90.4%) than LREEs (81.3%). Even after *B. pumilus* was freeze-dried, the adsorption amount of REEs and its preferential adsorption toward HREEs remained unchanged. Considering that FDP maintained its intact cell structure but without metabolic activity, the possibility of REE transport to the inside of cells could be excluded. This means that the adsorption sites should be external bacterial surface. Note that *B. pumilus* secretes EPS that can serve as adsorbents for REEs (Fig. S11) (40). However, our adsorption experiments indicate that EPS extracted from *B. pumilus* had a lower REE adsorption capacity (52.4%) than the control *B. pumilus* (86.8%), with a preferential adsorption of LREEs (55.3%) over that of HREEs (50.4%). The adsorption characteristics of EPS resembled those of G^−^ bacteria (e.g., *Porphyrobacter* sp. and A. caeni) that were isolated from the deposit profile. In contrast, the cell wall (CW) of *B. pumilus* displayed a very high affinity toward HREEs (69.2%) over LREEs (60.1%) with an appreciable adsorption efficiency for REEs (65.5%), resulting in a conspicuous leftward-inclined pattern across REE series. Thus, the CW, instead of EPS, was proposed to be the main adsorption location responsible for HREE and LREE fractionation on *B. pumilus*. According to the TEM and FTIR analyses, REEs were bonded to the phosphate group mainly on CW of *B. pumilus*. Note that compared to G^−^ bacteria, teichoic acids are the unique ingredients in the cell wall of G^+^ bacteria (40), and their content can account for up to 50% of the total dry weight of cell wall (41). This implies that the complexation of REEs with teichoic acids led to the enrichment of HREEs on G^+^ bacteria. To confirm this speculation, teichoic acids were separated from the CW of *B. pumilus* for further investigation. The absence of teichoic acid in the CW decreased the REE adsorption from 65.5% to only 17.8%, with a flat distribution pattern of the adsorbed REEs (i.e., no preferential HREE enrichment). Thus, teichoic acids were proposed to be responsible for the preferential adsorption of HREEs by the G^+^ bacteria.

## DISCUSSION

### Abiotic control of REE mineralization.

It is well known that under warm and humid climate conditions, weathering processes can give rise to the accumulation of REEs in the weathering crust, especially HREEs, relative to the bedrock of regolith-hosted REE deposits (7, 42, 43). Consistent with previous studies (44), both LREEs and HREEs were tremendously enriched at the top of B horizon in the Renju deposit. Note that the enrichment of HREEs is more significant than that of LREEs [e.g., most (LREEs/HREEs)_N_ < 1, Fig. 1] in the B horizon, despite that the bedrock displays remarkable depletion of HREEs when normalized with PAAS or average chondrite (45, 46). Such REE distribution characters were also observed in other typical regolith-hosted REE deposits, such as Zudong, Guposhan, and Huashan deposits in Southeast China and several deposits in Madagascar and Myanmar (7, 42, 43).

Granites, which contain weatherable REE-bearing minerals, are considered to be the main source of regolith-hosted deposits (7). In the Renju deposit, REE-bearing minerals in the bedrock mainly include titanite, apatite, allanite, monazite, xenotime, zircon, and secondary REE fluorocarbonates produced by deuteric hydrothermal processes (44). During weathering processes, titanite, allanite, and REE fluorocarbonates that are susceptible to weathering broke down in the lower B horizon, while apatite was severely weathered and disappeared in the upper B horizon (Fig. S2). Note that phosphates are generally considered to be strongly resistant to chemical weathering (47). However, apatite, the most common phosphate in granites, was found to be readily weathered by microorganisms (48), while phosphorous is an essential element for all life forms but usually limited in natural environments. For example, ectomycorrhizal fungi can activate and release elements from apatite, and these released elements can be accumulated in mycelia (49). In contrast, zircon is ubiquitous due to its strong resistance to weathering, while monazite and xenotime are preserved in the A horizon.

Dissolution of REE-bearing minerals and accumulation of those released REEs on clay minerals are the critical steps for the formation of regolith-hosted REE deposits, resulting in a dramatic change of REEs’ occurrence state from mineral phase to ion-exchangeable species. At the onset of weathering, the preferential dissolution of REE-bearing minerals (e.g., titanite, allanite, and REE fluorocarbonates) gave rise to the release of REEs into soil solution (8). The downward migration of weathering fluids resulted in a decrease of total REE content in the A horizon relative to that in the P horizon (Fig. 1) (50). With the increase of pH with depth, the dissolved REEs in weathering fluids were adsorbed and accumulated by clay minerals in the lower part of the regolith. This process led to the relative enrichment of total REEs in the B horizon compared to those in other horizons. The weathering-susceptible minerals, such as plagioclase, biotite, and amphibole in the bedrock, were decomposed and transformed to smectite/illite and then to kaolinite/halloysite (Fig. S3 and Table S1) (42). Illite and smectite occur mainly in the bottom of B horizon, far away from the main horizon of REE leaching, which makes the kaolinite and halloysite the dominant clay minerals controlling the enrichment of REEs (7). Though it is generally regarded that clay minerals are the predominant carriers of REEs in weathering profile, the adsorption of kaolinite and halloysite toward REEs did not display any selectivity (14). This suggests that adsorption of REEs by clay minerals cannot lead to fractionation between LREE and HREE while prominent HREE enrichment is the most important trait of regolith-hosted REE deposits in terms of scientific and economic interest (51). Meanwhile, the total REE content and (LREEs/HREEs)_N_ do not correlate with the distributions of kaolinite and halloysite (Fig. 1). This suggests that clay minerals are not the only factor responsible for the REE enrichment and fractionation and, instead, implies that some other factors may also have contributed to the REE mineralization. Given the wide distribution of microorganisms along the weathering profile (Fig. 2), and their ability in mineral dissolution (22) and REE adsorption (52), the effects of microorganisms on REE mineralization are discussed in the following sections.

### Correlations between REEs and the microbial community.

The microbial abundance and diversity in weathering crusts are enormous but are just beginning to be recognized (53). Recent advances in high-throughput sequencing technologies have eliminated the need for laboratory cultivation and/or isolation of individual species (54), revealing that the microbiome in the surface soil layer is highly diverse and comprises up to one quarter of Earth’s diversity (55). Although microbial diversity and REE distribution may vary greatly from profile to profile, the study of the relationships between microbial diversity and REE enrichment/fractionation along a complete profile is significant for understanding the microbial-accelerated REE mineralization process.

It is generally believed that the biomass and diversity of bacteria in surface soil are significantly higher than those in deep soil (56). In the Renju deposit, microbial biomass is high at 1 to 2 m but relatively low in the deeper part. It was found that MBC is significantly positively correlated with (LREEs/HREEs)_N_ ratio but uncorrelated with the content of REEs. Such correlations can be partly attributed to a scenario that microbes in the profile, as a whole, tend to facilitate REE fractionation considering their ability to dissolve REE-bearing minerals, as well as adsorb REEs (26, 34, 57), suggesting a remarkable effect of microorganism on REE mineralization. The bacterial diversity along the profile has a trend similar to that of REE fractionation, e.g., both decrease from the surface of weathering profile (~1 m) to the middle part of the B horizon (~25 m) but then increase in the lower part of the B horizon. Both the lowest bacterial diversity and (LREEs/HREEs)_N_ ratio occurred at ca. 25 m. Compared with the fungal diversity in the surface soils (0 to 10 cm) of the Xinfeng regolith-hosted REE deposit in Jiangxi Province, Southeast China (58), the diversity of fungi in the topsoil of the Renju deposit is relatively low. In contrast, soil samples at ca. 6 m with the highest fungal diversity have the highest REE content (Fig. S5). These results suggest that bacteria and fungi may affect REE mineralization through different mechanisms.

Given that dominant taxa generally have larger biomasses than nondominant taxa, dominant phyla/genera should have greater importance for REE enrichment/fractionation among the microbial community. All the dominant bacterial phyla (each with an average relative abundance of >1% across the profile) in the Renju deposit were also reported previously to prevail in the bacterial community of surface soil around an REE deposit in Jiangxi, China (27). Among them, *Proteobacteria* (46.9%), *Acidobacteria* (14.6%), *Actinobacteria* (9.01%), and *Firmicutes* (6.28%) are the most abundant. Many species of these phyla are known to be able to facilitate leaching, enrichment, and fractionation of REEs (34, 52, 59).

Many members of the two dominant genera *Acinetobacter* (5.06%) and *Pseudomonas* (1.25%) belonging to *Proteobacteria* were reported to be able to dissolve REE-bearing minerals, leading to the release of REEs (57, 60). Moreover, several strains of *Proteobacteria* have been found capable of enriching and fractionating REEs (23, 38, 59). *Pantoea* was found to enhance the release of P and REEs from apatite and enrich REEs by intracellular accumulation (61). Growth of methanotrophic microbe *Methylacidiphilum* isolated from volcanic mudpots has been shown to be strictly dependent on the presence of REEs (62). In Gram-negative bacteria, the oxidation of methanol is catalyzed by pyrroloquinoline quinone-dependent methanol dehydrogenase (63). In recent years, it was shown that REEs, especially LREEs, were essential as cofactors in XoxF methanol dehydrogenase in methylotrophic bacteria as well as *Pseudomonas* (64). Despite that there is no significant correlation between *Proteobacteria* and REEs in the Renju weathering profile, the above-mentioned complex interactions indicate that some members of this dominant bacterial phylum may play an important role in REE mineralization.

Although most operational taxonomic units (OTUs) of *Acidobacteria* are represented by uncharacterized order- and family-level groups, they are considered to have an oligotrophic (K-strategist) lifestyle, which facilitates their living in the low-nutrient environments in regolith-hosted REE deposits. In addition, many species affiliated with *Acidobacteria* have a metal-detoxification ability (65), raising the possibility that they are resistant to REE toxicity in the environment. A variety of strains of *Actinobacteria* were reported to secrete various organic acids and iron carriers that can leach REEs from bastnaesite and its bearing rocks, with a higher efficiency for HREEs than for LREEs (52). For *Firmicutes*, *Bacillus* affiliated with this phylum showed preferential adsorption toward HREEs (59). These findings can explain the significant correlations between (LREEs/HREEs)_N_ ratio and the concentrations of *Acidobacteria* and *Firmicutes*, indicating the potential contribution of these phyla to the fractionation of REEs (Fig. 3). *Planctomycetes*, *Chloroflexi*, and *Nitrospirae* are also significantly positively correlated with REE fractionation. Due to the complex effects of microorganisms on REE mineralization, these phyla may also contribute to REE enrichment and fractionation through other mechanisms such as mediating oxidation-reduction reactions and promoting rock dissolution and secondary mineral precipitation (66), which deserves further study. However, it is worth noting that the correlations between all the fractionation-related phyla and REE enrichment parameters are not significant (*P* > 0.05, detailed data not shown). This indicates that the microbial taxa that cause fractionation are not the dominant enrichers of REEs during the weathering process.

The dominant fungal phyla in the Renju deposit are *Ascomycota* (47.8%) and *Basidiomycota* (40.4%), which also predominate the fungal community in the tailing soils (0 to 10 cm) around Xinfeng REE deposit (58). Several genera belonging to *Ascomycota* are found to be correlated with REE contents. *Aspergillus* affiliated with *Ascomycota* is capable of dissolving phosphorus-bearing minerals and leaching and adsorbing REEs (67). Interestingly, we found that the relative abundance of *Aspergillus* in the Renju deposit is up to 8.89%, only next to *Malassezia* (27.4%). *Coniosporium*, with a relative abundance of 7.70%, is another dominant genus affiliated with *Ascomycota*. This genus is widely distributed on rock surface (18) and can accelerate granite weathering (68). Thus, although neither *Aspergillus* nor *Coniosporium* showed a significant correlation with the REE distribution along the profile, they have the potential to accelerate granite weathering and REE release, thereby promoting adsorption of REEs by other coexisting fungi.

Among the other dominant fungal genera, the concentration of *Trichoderma* is correlated with REE content. Some members of *Trichoderma* isolated from weathered rock surfaces were found to be capable of dissolving Ca-Mg-Zn silicates (69) and leaching REEs from rocks. Notably, many members of *Trichoderma* not only show a good tolerance to REEs but also can adsorb REEs both within and outside their cells (70). This implies a crucial role of *Trichoderma* in the migration and enrichment of REEs. Additionally, the concentrations of several other dominant fungal genera, including *Pyricularia*, *Microascus*, *Strelitziana*, *Tomentella*, *Phaeosphaeria*, *Pilatoporus*, *Entoloma*, and *Lentinus*, are also positively correlated with the REE content, highlighting their potential contributions to granite weathering and REE enrichment.

### Enrichment and fractionation of REEs by bacteria.

In the Renju profile, most of the isolated strains exhibited considerable adsorption toward REEs with an adsorption efficiency of >30% (Fig. 4), especially G^+^ bacteria (with an adsorption efficiency >75%). Additionally, several bacterial genera, which were reported with a high adsorption capacity of REEs (25, 52), also exist in the Renju profile with high abundance. For example, Pseudomonas aeruginosa is the first reported bacterium that can effectively adsorb La(III), opening the prelude to the study on REE adsorption by bacteria (71). In the Renju profile, *Pseudomonas* is one of the dominant bacterial genera with relative abundance of 1.25% (Fig. 2b). Bacillus pumilus and *Micrococcus* species strains were isolated from the studied profile, displaying adsorption efficiency of 76% to 90% and 67% to 93%, respectively. Through the batch experiments using model bacteria, Bacillus licheniformis showed a prominent adsorption capacity of Sm(III), i.e., 48 mg g^−1^ (dry weight) (72). *Micrococcus* sp. isolated from the Maoniuping REEs deposit, Sichuan Province, China scavenged REEs with an efficiency of 50% to 80% across the series (52). The aforementioned phenomena indicate that bacteria should play a vital role in REE enrichment during the formation of deposit.

Note that, among the isolated strains in the Renju profile, only *Micrococcus* sp. and *B*. *pumilus* that are G^+^ bacteria showed preferential adsorption of HREEs over LREEs, while all the G^−^ bacteria did not give rise to REE fractionation. Such distinct REE fractionation characters have been reported in the literature (72, 73). On the surface of G^+^ bacteria Bacillus subtilis, the accumulation of HREEs, especially Tm(III), Yb(III), and Lu(III), was much higher than that of LREEs (59). In contrast, the G^−^ bacteria *Roseobacter* sp. AzwK-3b did not show obvious adsorption selectivity between LREEs and HREEs (74).

In terms of the adsorption mechanism, Ngwenya et al. found that REEs were primarily bound to the phosphate sites and subsequently to the carboxylate sites on the surface of bacterial cells by using extended X-ray absorption fine structure spectra (38). However, the contribution of phosphate and carboxyl groups to REE adsorption relied on the surrounding pH. At lower pH range, REEs mainly interacted with phosphate groups, while at around pH 2 and above, carboxyl groups also contributed to the uptake of REEs (34). Due to the pH range of 4.3 to 7 in the Renju profile, both phosphate and carboxyl groups are active sites responsible for the REE adsorption on bacterium surface, which was revealed by elemental mappings and FTIR spectra (Fig. 5 and 6).

However, this finding could not account for the enrichment of HREEs on merely strains affiliated with *Actinobacteria* and *Firmicutes*, considering that all the investigated bacteria have carboxyl and phosphate groups. According to the adsorption characters of REEs on different components of native *B. pumilus*, two adsorption sites are proposed. On the one hand, EPS that are secreted by bacteria and consist of polysaccharides, proteins, nucleic acids, and lipids (75) are able to capture REEs (76). But compared to *B. pumilus*, the secreted EPS adsorbed REEs without obvious fractionation, and the adsorption efficiency was much lower. This is consistent with previous observations (59) and excludes the possibility that EPS serve as the main component for HREE accumulation. On the other hand, among the living organisms, bacteria have the highest ratio of surface area to volume (77). Thus, cell wall is another adsorption sites via the complexation of metal ions with the functional groups (78). In this study, REEs were immobilized on the cell wall of *B. pumilus* with a high efficiency, displaying higher enrichment of HREEs than of LREEs. This suggests that the cell wall of G^+^ bacteria was responsible for the adsorption and fractionation of REEs.

Our further investigations showed that teichoic acid on the cell wall of *B. pumilus* is the main active site for its selective adsorption of HREEs. This acid has phosphate groups, which are known to preferentially bind with HREEs (74). Teichoic acid is the unique cell wall component of G^+^ bacteria (79), thereby giving a reasonable explanation for the selective adsorption of HREEs on the studied G^+^ bacteria rather than the G^−^ ones. As an important component of cell wall, teichoic acid can account for up to 50% of the total dry weight of cell wall of some G^+^ bacteria. In addition, teichoic acid was even reported to be able to transform the adsorbed REEs into monazite (80). These characteristics of teichoic acid are beneficial for it to exert an important role in selective adsorption of HREEs on G^+^ bacteria.

**Concluding remarks.** Regolith-hosted REE deposits are dominantly distributed in subtropical regions with strong microbial activities. For the first time, our study reports the community composition and diversity of microorganisms in a typical regolith-hosted REE deposit and illustrates the remarkable enrichment and fractionation of REEs by the isolated bacteria from the studied deposit. Many microbial phyla and genera are correlated with REE enrichment and fractionation. Both fungi and bacteria promote the enrichment of REEs, whereas, compared to fungi, bacteria play a prominent role in the fractionation between HREEs and LREEs. As demonstrated by the controlled adsorption experiments, bacteria adsorb REEs through the complexation with surface carboxyl and phosphate groups. G^+^ bacteria that have teichoic acids in the cell walls show a preferential adsorption of HREEs, contributing to the enrichment of HREEs.

So far, most regolith-hosted REE deposits are located primarily in the area underlain by the Jurassic-Cretaceous granites and developed in areas with subtropical climate and exuberant microbial activities (7). Given the high abundance of microorganisms in supergene environments and a long geological time scale of deposit formation, the cumulative contribution of microorganisms in the genesis of regolith-hosted REE deposits should be appreciable. Thus, we can reasonably speculate that microorganisms may also play an important role in the formation of exogenic deposits other than REEs. Note that several identified fungi and bacteria strains in the deposit profile have high potentials not only in enriching and fractionating REEs but also in leaching REEs from the REE-bearing minerals. This is of important enlightening significance for the microbiological extraction of REE resources.

## MATERIALS AND METHODS

### Study site and sampling.

The Renju REE deposit that is located in Renju Town, Meizhou City, Guangdong Province currently has 20,467 tons of rare earth oxides (REOs) with an average grade of 0.172 wt% REOs (44). The deposit is hosted in the weathering crust of the Jurassic Renju granitic pluton with an outcrop area of 29.3 km^2^. This pluton represents products of extensive Mesozoic magmatic activities in Southeastern China.

The pluton consists of biotite granites, granite porphyries, and quartz diorites. Northeast-trending and northwest-trending faults are developed mainly in this area. The pluton intrudes Cretaceous and Cambrian sedimentary successions. The pluton intrudes Cretaceous and Cambrian sedimentary successions. The distribution of the orebody developed in the weathered crust of the biotite granites is controlled mainly by topography, and orebodies occur as crescent lens with an average thickness of 5.6 m.

The Renju REE deposit is located in a region with moderately undulating landforms, covered by vegetation and trees. The terrain is dominated by a small (~35 m) hill with a <25° slope. The elevation varies from 250 to 350 m above sea level. The climate in this region is subtropical and influenced by the East Asian monsoon, with an average annual temperature of approximately 25°C and an average annual rainfall of 1,500 to 2,000 mm (14). The perennial moist and warm climate provide ideal conditions for intense chemical weathering and microbial activity.

A drill hole penetrated a granite weathering profile of the Renju deposit (24°59′5″ N; 115°50′23.6″ E). Drill core samples were collected using a custom-made double-tube drilling system comprising a stainless-steel outer tube equipped with a diamond drill bit and an inner PVC tube (8). Nine samples were collected at 1, 2, 4, 6, 10, 20, 25, 40, and 45 m of the deposit profile, respectively. Each sample was divided into three parts. One part was sealed in zip bags, dried at room temperature, and ground to fine powder with a rotary disk mill, followed by the analyses of pH and contents of major elements and REEs. Another part used for measurement of MBC and cultivation of microbial isolates was packed in sterilized centrifuge tubes (50 mL), stored at 4°C in a sterile refrigerator, and processed within 3 days after collection. The remaining fraction used for characterizing microbial diversity and community composition was packed in sterilized centrifuge tubes (50 mL) and then frozen at −18°C in the refrigerator before use.

### pH and element analysis.

The pH of each sample was measured by equilibrating 10.00 g of sample powder (20 mesh) with 25 g deionized water in a capped beaker for 8 h. Then, the liquid was collected by filtration. The pH of liquids was measured using a Mettler-Toledo FiveEasy Plus pH meter with a precision better than 0.05.

Before the element analysis, all the samples were calcined at 900°C for 90 min to remove organic matter and carbonate. Then, about 0.50 g of each treated sample was weighed, mixed with 4.00 g Li_2_B_4_O_7_, and fused into glass discs at 1,200°C. The major elements were measured from the glass discs using a Rigaku ZSX100e X-ray fluorescence spectrometer (XRF, Rigaku, Japan). The analytical precision of major element contents was better than 1%. The chemical index of alteration (CIA) of the weathering profile is calculated by CIA = [Al_2_O_3_/(Al_2_O_3_ + CaO* + Na_2_O + K_2_O)] × 100, where CaO* is the amount of CaO incorporated in the silicate fraction of the rock (81).

Approximately 200 g of bulk samples was dried at 105°C for 3 h and then baked at 550°C for 3 h to eliminate organic materials. Then, about 0.04 g of each solid sample was digested with a mixture of HNO_3_–HF–HClO_4_ in a closed Teflon beaker and heated in an oven at 190°C for 40 h. The digested samples were dried at 100°C and then redigested with a mixture of HNO_3_–HF–HClO_4_ at 190°C for 2 days. The redigested samples were dried at 100°C, dissolved using HNO_3_ until no residue remained, and then further diluted with 2% HNO_3_ for trace element measurements (82). Rh (rhodium) was added to each sample as an internal standard to calibrate the drift of the instrument during the measurement. The analyses were carried out with an inductively coupled plasma-mass spectrometer (ICP-MS; Thermo iCAP Qc, ThermoFisher Scientific, USA) to an accuracy exceeding 3% relative standard deviations. Several USGS (United States Geological Survey) and Chinese rock and sediment standards (GSR-2, GSR-3, GSD-09, GSD-11, SARM-4, W-2, and AGV-2) were used as external standards for quality control.

### Mineral compositions and petrographic observations.

The mineral compositions of the bedrock and regolith were analyzed using a Bruker D8 advance X-ray diffractometer (XRD). The scan range (2θ) was between 3° and 80° at a scanning speed of 3° min^−1^ with Cu Kα radiation (40 mA and 40 kV). The XRD patterns were analyzed using JADE 6.0 software to obtain quantitative mineral composition data.

Thin sections of bedrock and weathering crust were prepared for detailed mineralogical and petrographic analysis to identify paragenesis of minerals, and the target minerals were marked. Subsequently, the thin sections were carbonized for scanning electron microscopy analysis (SEM) of mineral composition and mineral weathering characteristics using a Hitachi SU8010 SEM equipped with an energy-dispersive X-ray spectrometer (EDS) at 15 kV. Electron probe analysis was carried out to test the mineral chemical composition of the target minerals by a JEOL JXA-8230 electron microprobe. The acceleration voltage was 20 kV, the current was 20 nA, and the beam spot size was 1 μm.

The morphology, composition, phase, and structure of submicron REE-bearing minerals in weathering crust were studied by high-resolution transmission electron microscopy (HRTEM), X-ray energy-dispersive spectroscopy (EDS), high-angle annular dark-field scanning transmission electron microscopy (HAADF-STEM), and selected-area diffraction (SAED) by an FEI Talos F200S instrument operating at 200 kV.

### Measurement of microbial biomass carbon.

Microbial biomass carbon was measured by chloroform fumigation extraction method (30). Moist soil at 40% of its water-holding capacities and containing the equivalent of 50 g oven-dry soil was fumigated with ethanol-free CHCI_3_ for 24 h at 25°C in sealed desiccators containing water and soda-lime. The fumigant was then removed and the soil was extracted with 0.5 M K_2_SO_4_. A nonfumigated control was extracted under the same conditions at the time fumigation commenced. The extracts were analyzed by a total organic carbon analyzer (Vario TOC select, Elementar, Germany).

### DNA extraction, PCR amplification, and Illumina HiSeq sequencing.

DNA was extracted from each sample using a DNA extraction kit (mCHIP, China) according to the manufacturer’s instructions. DNA integrity and purity were monitored on 1% agarose gels. DNA quality was determined using the NanoDrop 2000 spectrophotometer (Thermo Scientific, USA). The PCR amplification is described in Text S2. The PCR products were purified using silica-based columns and later were used to generate the sequencing libraries using NEBNext Ultra DNA library prep kit for Illumina (New England Biolabs, USA) following the manufacturer’s recommendations. The library quality was assessed on the Qubit 2.0 fluorometer (Thermo Scientific, USA) and Agilent bioanalyzer 2100 system (Agilent, USA). The library was sequenced on an Illumina HiSeq 2500 platform (250 bp paired-end reads mode). The diversity and composition of bacterial and fungal communities in the regolith were assessed by sequencing the V4 hypervariable region of 16S rRNA gene and the internal transcribed spacer region ITS2 (83), respectively.

### Bioinformatic analysis.

Raw sequencing data were treated with Mothur (84) and QIIME (85). Chimeras and reads with ambiguous bases were removed, and sequences with average quality score of <25 were discarded. Then, the remaining good-quality sequences were assigned to each sample by specific barcodes and clustered into operational taxonomic units (OTUs) at 97% identity threshold. The OTU taxonomy synthesis information is described in Text S3. Resampling was performed to randomly select the same number of sequences (sequence number) per sample. The α-diversity was estimated by two widely used indices (Chao1 for richness and Shannon for combination of richness and evenness) based on the resampled sequences. Both indices were calculated using QIIME (V1.9.1). The sequences analyzed in this paper have been deposited in the NCBI database (accession no. PRJNA728215).

### Bacteria isolation and REE adsorption experiments.

R2A was used as the separation culture medium. R2A had the following composition (in g L^−1^ of ultrapure water): yeast extract, 0.50; proteose peptone, 0.50; casamino acids, 0.50; glucose, 0.50; soluble starch, 0.50; Na-pyruvate, 0.30; K_2_HPO_4_, 0.30; MgSO_4_**·**7H_2_O, 0.05; agar, 15.00 (pH 6.9 to 7.1). Soil samples from different depths of the profile were collected and stored at 4°C in a sterile refrigerator. The samples were then air-dried within 3 days after collection. Dry soil (1 g) was stirred for 5 min with 100 mL of sterile ultrapure water in a conical flask. After 30 min, the serial dilutions of supernatant were prepared with the suspension (86). Over the surface of solidified agar plates, 0.1-mL samples of the proper dilution were spread with a sterile glass rod. The plates were incubated at 28°C for 5 to 7 days. Then, different colonies were isolated. The colonies obtained were classified according to the 16S rRNA gene sequences.

The adsorption experiments of REEs on bacteria were carried out using nine bacteria strains isolated from the Renju weathering profile. All the bacteria were cultivated in Luria-Bertani (LB) medium at 25°C for 24 h. LB had the following composition (in g L^−1^ of ultrapure water): yeast extract, 5.00; tryptone, 5.00; NaCl, 10.00 (pH 6.9 to 7.1). They were then separated from the growth media by centrifugation at 3,000 rpm for 15 min and washed five times with 1.0 mmol L^−1^ NaCl solution. To start the adsorption experiment, an REE standard solution containing all the REEs (except Pm) was added to the bacterial suspensions (1 g L^−1^). The initial concentration of each REE in the suspension was fixed at 1 ppm. The pH was adjusted to 5 by the addition of HNO_3_ or NaOH solution (0.1 mol L^−1^). The samples were gently shaken for 60 min, the predetermined time for achieving adsorption equilibrium (59). For each reaction system, the aqueous phase was separated from the bacteria by filtration using hydrophilic polytetrafluoroethylene (PTFE) filter (0.45 μm). The filtrate was acidified with HNO_3_ to a final concentration of 2% for the determination of aqueous REE concentration using an ICP-MS (Thermo iCAP Qc, ThermoFisher Scientific, USA). The precision of ICP-MS measurement was better than 3%.

### Analytical methods.

A high-resolution transmission electron microscope (TEM; Talos F200S, FEI, USA) equipped with Super-X X-ray spectroscopy operated at 200 kV was employed for high-resolution imaging and compositional analysis. The morphology of *B. pumilus* before or after Yb(III) adsorption was observed by TEM (80). The cell suspension was centrifuged and washed three times with NaCl solution (1.0 mmol L^−1^).

The EDS mapping analyses of the *B. pumilus* before and after Yb(III) adsorption were carried out to reveal the distributions of elements (e.g., C, N, O, P, and Yb) in the cells. Oriented samples were embedded in epoxy resin and dried at 100°C for 3 h. Then, ultrathin sections with a thickness of ca. 75 nm were sliced with a diamond knife using a Lecia EM UC7 ultramicrotome. The sections were placed on carbon-coated copper microgrids for TEM and high-angle annular dark-field scanning transmission electron microscopy (HAADF-STEM) with an FEI Talos F200S microscope at an accelerating voltage of 200 kV.

The change of surface functional groups on *B. pumilus* before and after Yb(III) was analyzed using a Fourier-transform infrared (FTIR) spectrometer (Vertex 70, Bruker, Germany). Sixty-four scans were collected for each measurement in the spectral range of 4,000 to 400 cm^−1^ with a resolution of 4 cm^−1^.

### Preparation of different bacterial cell components.

*B. pumilus* isolated from the Renju weathering profile was cultivated in LB medium at 25°C for 24 h, then separated from the growth medium by centrifugation at 3,000 rpm for 15 min, and washed five times in NaCl solution (1.0 mmol L^−1^). The bacteria were then freeze-dried into a freeze-dried powder (FDP). To isolate the EPS of *B. pumilus*, 50 mL of the bacteria suspension was taken and sonicated at 40 W for 2 min. The supernatant was separated from the bacteria suspension by centrifugation at 20,000 × *g* and 4°C for 20 min. The supernatant obtained was then centrifuged at 10,000 × *g* and 4°C for 15 min (87). EPS solution was stored at −18°C before use. Cell walls were prepared from exponential-phase cultures of *B. pumilus* by differential centrifugation after disruption by sonication at 400 W for 50 min (88). The walls were washed three times in NaCl solution (1 mmol L^−1^), washed five times in water, and then freeze-dried. The teichoic acid-free (TAF) wall was prepared from the corresponding cell walls by the treatment with 5% (wt/vol) trichloroacetic acid at 35°C for 8 h, which has been proved to remove 95% of the teichoic acid but leave the peptidoglycan intact (89). The walls were washed free of trichloroacetic acid by repeated resuspensions and centrifugations from water and freeze-dried. The procedure for REE adsorption on control *B. pumilus* and its freeze-dried power and different components was the same as that for the adsorption experiments of REEs on bacteria.

### Statistical analysis.

The correlations between the community composition or microbial diversity and REE enrichment or fractionation were analyzed by Pearson correlation analysis. The two-tailed test was used to check for significance. All statistical analyses were performed with SPSS software (V23.0). Prism software (V8.0.1) was used to draw heat maps.

### Data availability.

The sequence analysis results in this paper have been deposited in the NCBI database (accession no. PRJNA728215). We declare that all other relevant data are available within the article and the supplemental material.
