# Supplementary material for: Microorganisms Accelerate REE Mineralization in Supergene Environments
Source: Appl Environ Microbiol. 2022 Jun 16;88(13):e00632-22. doi: 10.1128/aem.00632-22 (PMC9275249; doi:10.1128/aem.00632-22)
Supplement: Supplemental file 1 — Texts S1 to S3, Fig. S1 to S12, and Tables S1 to S5. Download aem.00632-22-s0001.pdf, PDF file, 1.5 MB [file aem.00632-22-s0001.pdf]

Supporting information for Microorganisms accelerate REE mineralization in supergene environments by Li *et al.*

TEXT S1 Texture, color and mineral composition of the weathering profile in Renju REE deposit

According to the texture, color, and mineral composition, the whole weathering profile is divided into three horizons with increasing CIA, including the completely weathered horizon (A horizon, CIA > 95); a semi-weathered horizon (B horizon, CIA = 60-95); and fresh bedrock (P horizon, CIA < 60).

The A horizon with CIA > 95 shows a red color and loose structure, containing a small amount of pores. The A horizon is mainly composed of supergene minerals with some rounded residual quartz grains, whereas primary feldspar and biotite almost disappear. Kaolinite and halloysite are the main supergene minerals, taking up 33%–71% of the total content in A horizon.

The B horizon with CIA values between 60 and 95 shows a mottled red and white color, which is probably induced by heterogeneous distribution of Fe (hydr)oxide minerals in a clay mineral matrix. The clay minerals in this horizon gradually change from kaolinite/halloysite to smectite/illite, with the contents of smectite/illite gradually increasing to 49% from top to bottom.

The P horizon, CIA < 60 is a fine-grained (0.5–2.0 mm) equi-granular quartz diorite that is composed mainly of plagioclase, K-feldspar, quartz, biotite, and amphibole. It also includes a small amount of chloritized biotite and amphibole.

TEXT S2 Procedure of PCR

16S rRNA gene and ITS2 region of bacteria and fungi were amplified using specific primers (i.e., 16S: 515F and 806R; ITS3-F and ITS4R) with a 12 bp barcode. Primers were synthesized by Invitrogen (Carlsbad, CA, USA). PCR reactions, containing 25 μL 2x Premix Taq (Takara Biotechnology, Dalian Co. Ltd., China), 1 μL each primer (10 mmol L^{-1}) and 3 μL DNA (20 ng μL^{-1}) template in a volume of 50 μL, were carried out by thermocycling with the following program: 5 min at 94°C for initialization, 30 cycles of 30 s denaturation at 94°C, 30 s annealing at 52°C, and 30 s extension at 72°C, followed by 10 min final elongation at 72°C. Three replicates per sample and each PCR product of the same sample were mixed, while the PCR instrument was BioRad S1000 (Bio-Rad Laboratory, CA). The length and concentration of the PCR products were detected by 1% agarose gel electrophoresis. Samples with a bright band can be used for further experiments. PCR products were mixed in equi density ratios according to the GeneTools Analysis Software (Version4.03.05.0, SynGene). The mixture of PCR products was purified with EZNA Gel Extraction Kit (Omega, USA).

TEXT S3 OTU statistics

Quality filtering on the paired-end raw reads was performed under specific filtering conditions to obtain the high-quality clean reads according to the Trimmomatic quality control process. At the same time, sequences were assigned to each sample based on their unique barcode, after which the barcodes were removed to obtain the paired-end clean reads. Paired-end clean reads were merged using FLASH according to the relationship of the overlap between the paired-end reads. When at least 10 of the reads overlap the read generated from the opposite end of the same DNA fragment, the maximum allowable error ratio of the overlap region of 0.2, and the spliced sequences were called raw tags. Quality filtering on the spliced sequences were performed under the Trimmomatic software to obtain the effective clean tags.

Sequence analysis was performed by using Usearch software. Sequences with $\geq$ 97% similarity were assigned to the same OTU. Representative sequence for each OTU was screened for further annotation. It is generally accepted that the singleton OTU is obtained by sequencing errors, or from chimeras generated during PCR. So the singleton OTUs were removed using Usearch after OTU cluster, and then the chimera sequences were detected and removed using the UCHIME de novo algorithm.

For each representative sequence, the GreenGene (for 16S, chloroplast and mitochondria) and Unite (for ITS) databases were used based on RDP classifier algorithm and the assign_taxonomy.py script in Qiime to annotate taxonomic information. The confidence threshold was set to default 0.5. The OTU and its Tags were removed, because they were annotated as chloroplasts or mitochondria (16S

amplicons) and could be annotated to the kingdom level. Thus the OTU taxonomy synthesis information was got for the alpha diversity analysis.

A total of 1,519,236 raw reads of 16S rRNA were obtained from the Illumina HiSeq sequencing analysis. The number of reads per sample ranged from 38,597 to 80,348. After quality filtering, denoising, and removal of potential chimeras and non-bacterial sequences, all effective sequences were distributed into 48,538 operational taxonomic units (OTUs) with a sequence similarity of 97%.

Equally, the raw reads of samples resulted in 4,122,528 of ITS averaging 152,686 for each sample. The number of reads per sample ranged from 57,963 to 415,987. After a series of data processing, the high-throughput sequencing of fungi resulted in 898,516 effective sequences averaging 33,278 sequences for each sample, with the average length of the 276 bp. A total of 4,047 OTUs determined at 97% sequences similarity were identified across the samples.

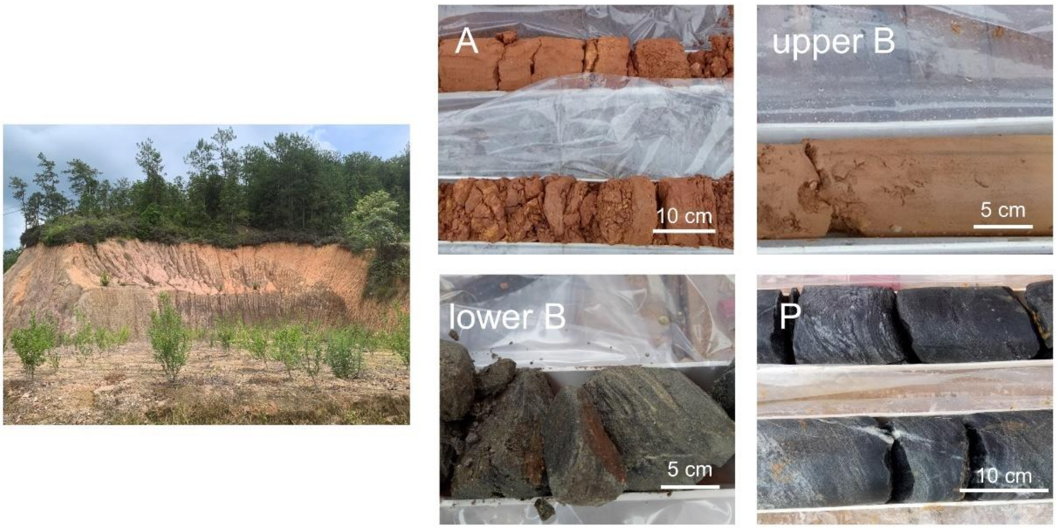

FIG S1 The photographs of the Renju weathering profile and the corresponding weathering stages. The weathering profile is divided into the A horizon (i.e., topsoil and complete weathering layer), the B horizon (i.e., incompletely weathered layer) and the P horizon (i.e., weathering front and bedrock).

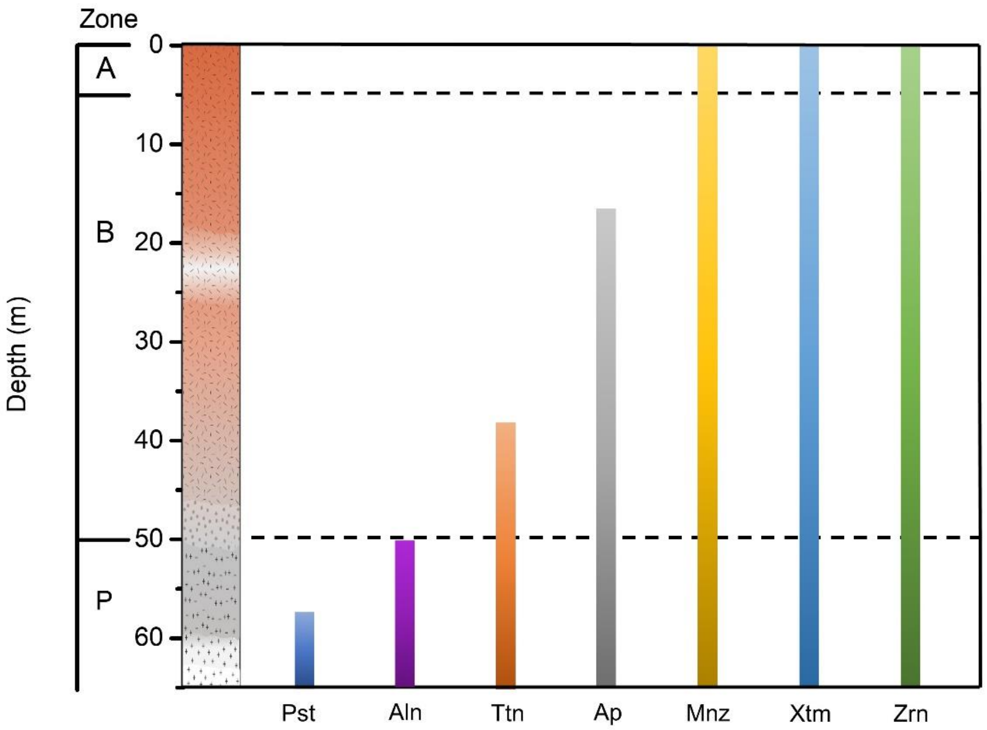

FIG S2 Distribution horizon of REE-bearing mineral in the Renju weathering profile. Pst = parisite; Aln = Allanite; Ttn = titanite; Ap = apatite; Mnz = monazite; Xtm = xenotime; Zrn = zircon.

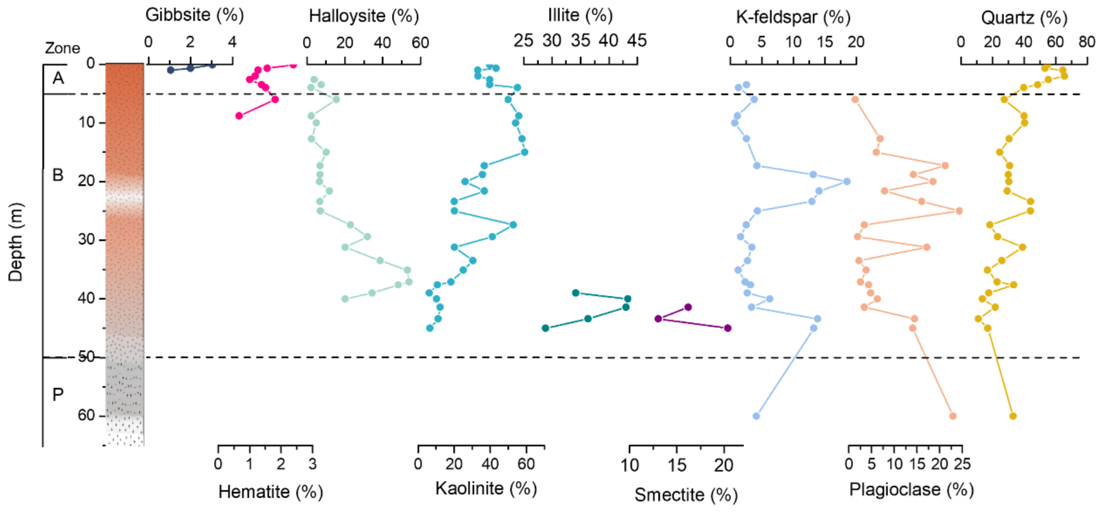

FIG S3 Depth-variations in the content of minerals in the Renju deposit profile.

The minerals mainly include gibbsite, hematite, halloysite, kaolinite, illite, smectite, K-feldspar, plagioclase and quartz.

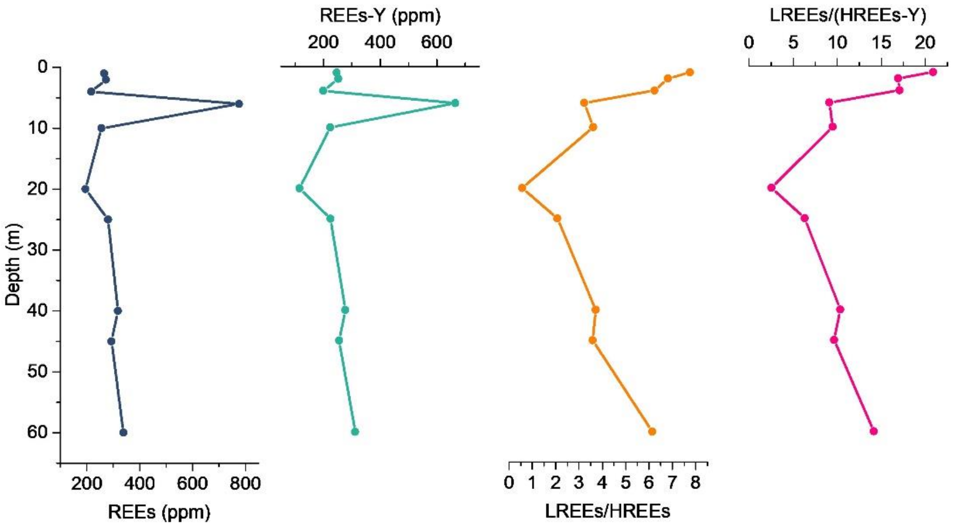

FIG S4 Depth-variations of REEs, REEs-Y, LREEs/HREEs and LREEs/(HREEs-Y) in the Renju deposit profile.

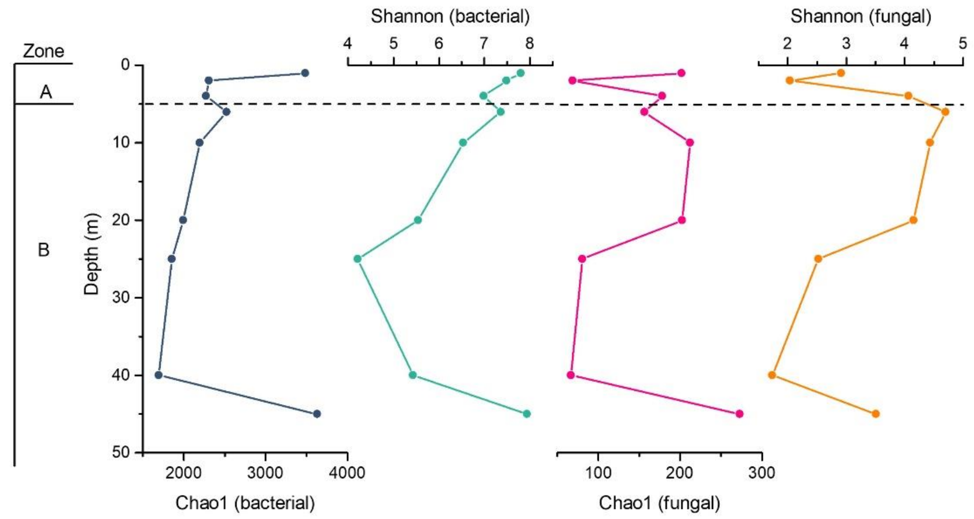

FIG S5 α-diversities of microbial communities in the Renju deposit profile. Two α-diversity indices, Chao1 and Shannon index, are evaluated.

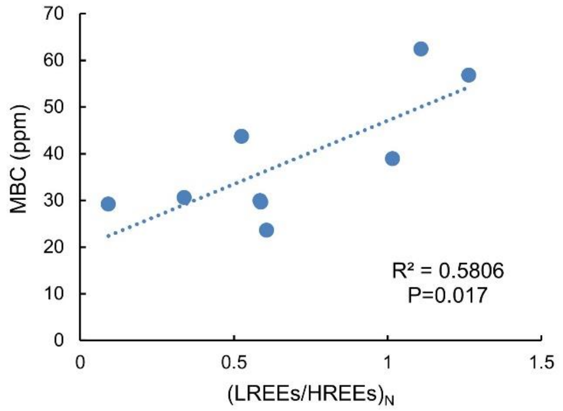

FIG S6 The correlation between (LREEs/HREEs)$_N$ ratio and the total microbial biomass carbon (MBC) concentration in the Renju deposit profile.

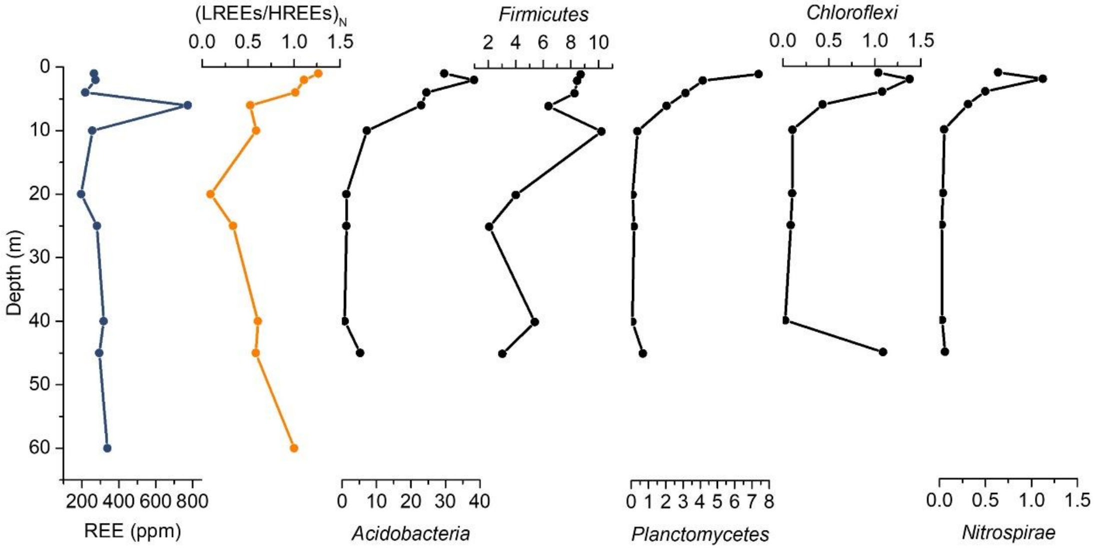

FIG S7 Depth-variations of the relative abundances (%) of bacterial phyla with significant correlations to REE fractionation.

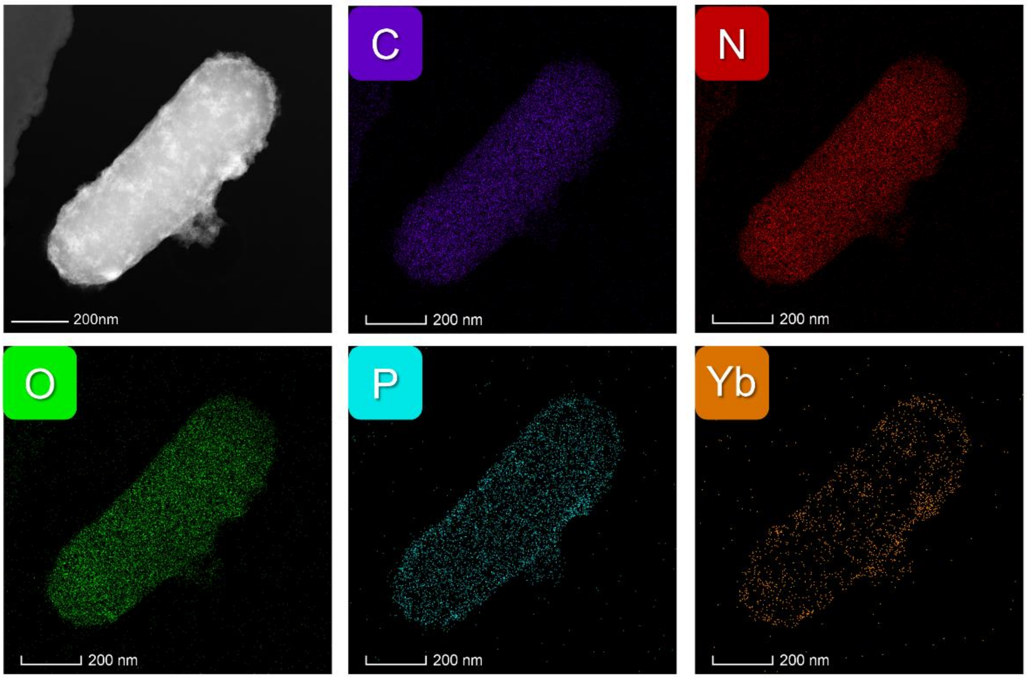

FIG S8 High-angle annular dark-field scanning transmission electron microscopy (HAADF-STEM) image and the corresponding element mappings of the *B. pumilus* after ytterbium (Yb(III)) adsorption. C, N and O are evenly distributed within bacterial cell, while high concentrations of Yb and P are observed along the cell wall.

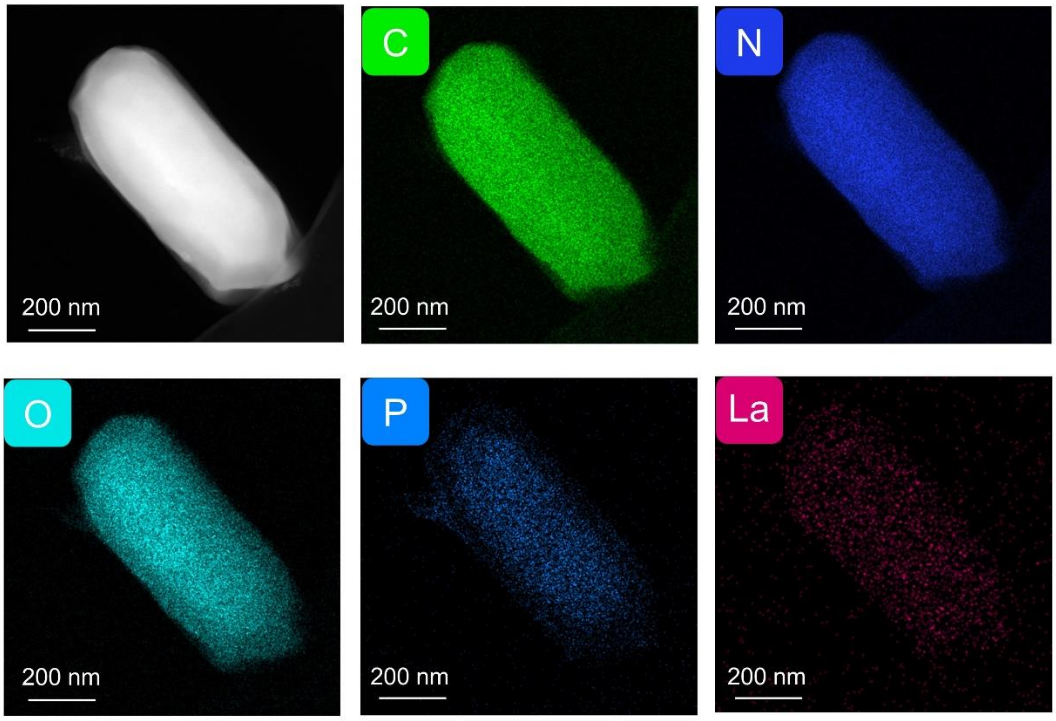

FIG S9 High-angle annular dark-field scanning transmission electron microscopy (HAADF-STEM) image and the corresponding element mappings of the *B. pumilus* after lanthanum (La(III)) adsorption.

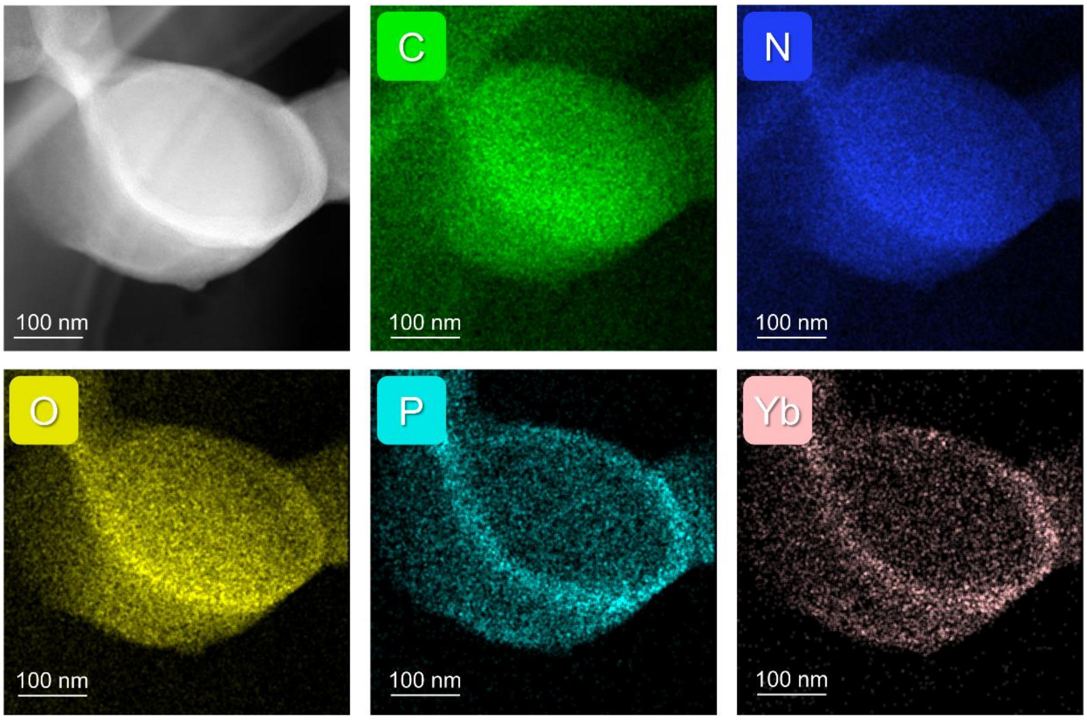

FIG S10 HAADF-STEM image and the corresponding element mappings of the cross-section of *B. pumilus* after ytterbium (Yb(III)) adsorption. C, N and O are evenly distributed within bacterial cell, while high concentrations of Yb and P are observed along the cell wall.

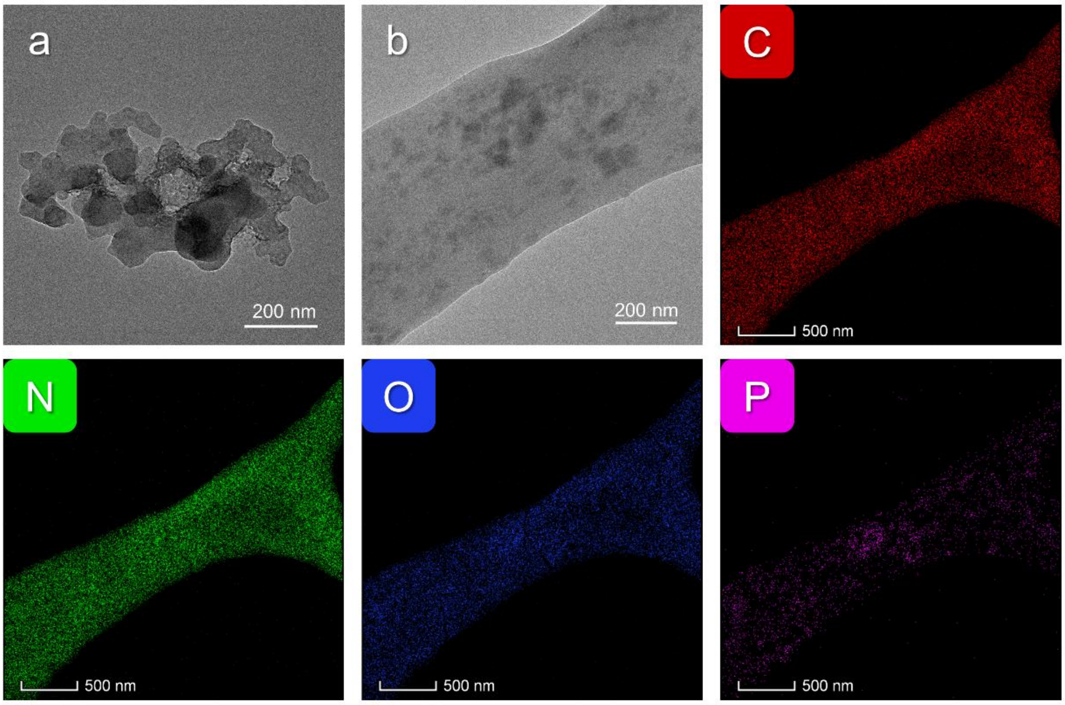

FIG S11 TEM images of EPS and the element mappings of C, N, O and P. (**a**) and (**b**) illustrate the complex structure of the extracellular polymeric substances (EPS) on which extracellular precipitates can be formed (1). C, N and O are evenly distributed within EPS, while P is preferentially distributed as agglomerate.

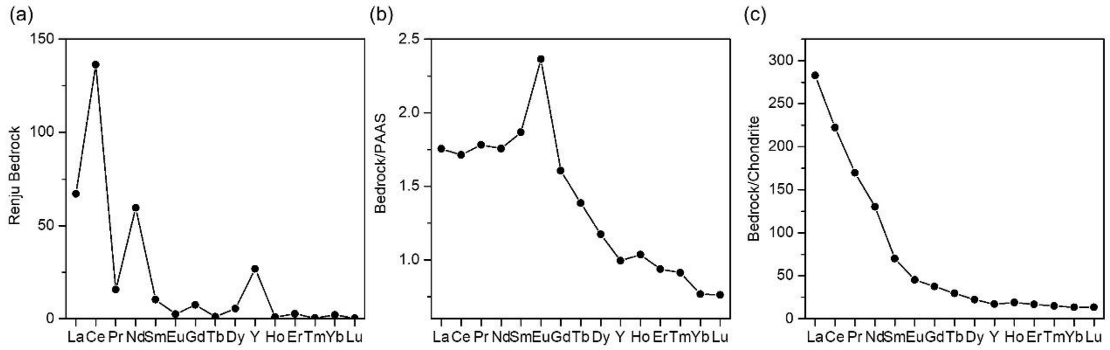

FIG S12 REE pattern in the Renju bedrock. (**a**)REE pattern in bedrock, (**b**) normalized by average chondrite, (**c**) normalized by PAAS.

TABLE S1 Relative abundances of minerals along the Renju weathering profile (wt.%).

Sampling Site	Gibbsite	Hematite	Kaolinite	Halloysite	Quartz	Plagioclase	K-feldspar	Illite	Smectite	Chlorite	Biotite	Amphibole
1 m	1	1	33		65							
2 m		1	33		66							
4 m		2	55	2	40		1					
6 m		2	50	15	28	1	4					
10 m			54	5	40		1					
20 m			26	7	30	19	19					
25 m			20	7	44	24	4					
40 m			10	20	14	6	6	43				
45 m			6		17	14	13	29	20			
60 m					10	53	4			10	10	14

TABLE S2 Variations in the content of each rare earth element with depth in the Renju weathering profile (ppm).

	1 m	*SE	2 m	SE	4 m	SE	6 m	SE	10 m	SE	20 m	SE	25 m	SE	40 m	SE	45 m	SE	60 m	SE
La	22.1	0.18	38.1	0.83	18.4	0.43	170	1.25	65.4	1.27	17.1	0.20	32.5	0.14	55.6	0.22	52.4	0.29	67.0	0.25
Ce	191	4.09	158	3.00	146	1.36	201	3.19	50.5	0.80	25.0	0.41	111	4.15	121	0.14	108	0.36	136	1.21
Pr	3.95	0.07	7.85	0.15	4.14	0.03	40.1	0.09	15.3	0.19	4.49	0.06	7.25	0.06	13.3	0.06	12.5	0.05	15.7	0.15
Nd	14.4	0.33	28.0	0.58	15.6	0.21	150	0.47	56.9	1.00	17.5	0.38	30.1	0.34	49.4	0.31	46.1	0.12	59.5	0.47
Sm	2.60	0.03	5.11	0.13	3.01	0.01	27.2	0.14	10.3	0.18	5.31	0.09	6.65	0.03	9.26	0.02	8.87	0.03	10.4	0.13
Eu	0.39	0.00	0.53	0.02	0.36	0.01	5.08	0.03	1.97	0.01	0.64	0.01	1.36	0.02	1.89	0.03	1.74	0.03	2.55	0.04
Gd	2.42	0.08	4.03	0.15	2.63	0.10	21.9	0.26	7.80	0.16	6.87	0.11	6.91	0.11	7.89	0.11	7.78	0.11	7.48	0.14
Tb	0.47	0.02	0.65	0.01	0.48	0.02	3.39	0.05	1.10	0.01	1.62	0.02	1.26	0.01	1.28	0.01	1.24	0.00	1.07	0.02
Dy	2.98	0.14	3.66	0.12	2.98	0.22	19.5	0.11	6.26	0.09	11.8	0.15	8.30	0.07	7.24	0.08	7.31	0.12	5.50	0.08
Ho	0.69	0.04	0.79	0.05	0.69	0.05	3.99	0.03	1.22	0.01	2.75	0.04	1.93	0.02	1.47	0.03	1.46	0.02	1.03	0.01
Er	2.17	0.16	2.39	0.16	2.15	0.15	11.0	0.16	3.26	0.03	9.00	0.11	6.40	0.06	4.11	0.03	4.07	0.09	2.67	0.04
Tm	0.36	0.03	0.38	0.02	0.33	0.03	1.63	0.02	0.48	0.01	1.50	0.02	1.14	0.01	0.60	0.01	0.59	0.01	0.37	0.00
Yb	2.34	0.12	2.58	0.17	2.08	0.16	10.2	0.12	2.98	0.04	10.0	0.09	8.31	0.08	3.62	0.06	3.53	0.04	2.16	0.02
Lu	0.39	0.02	0.44	0.03	0.33	0.03	1.56	0.02	0.47	0.01	1.57	0.02	1.36	0.00	0.58	0.01	0.55	0.01	0.33	0.01
Y	18.8	1.05	20.3	1.08	19.0	1.65	111	0.36	32.0	0.41	80.2	0.71	55.8	0.29	40.5	0.27	37.4	0.40	26.9	0.23

*SE: Standard error.

TABLE S3 Variations in the concentration of microbial biomass carbon with depth in the Renju weathering profile (ppm).

	1 m	2 m	4 m	6 m	10 m	20 m	25 m	40 m	45 m
1	60.8	62.5	40.9	43.1	35.8	32.3	28.5	24.9	27.5
2	55.7	60.9	38.3	46.5	30.5	28.0	33.0	22.4	30.7
3	54.1	64.0	37.8	41.6	22.6	27.6	30.5	23.7	31.7
Average	56.8	62.5	39.0	43.8	29.6	29.3	30.7	23.6	30.0
*SE	2.02	0.89	0.95	1.44	3.86	1.51	1.31	0.72	1.26

*SE: Standard error.

TABLE S4 Relative abundances (%) of dominant phyla and genera along the Renju weathering profile.

Bacteria phyla	1 m	*SE	2 m	SE	4 m	SE	6 m	SE	10 m	SE	20 m	SE	25 m	SE	40 m	SE	45 m	SE	Average
Proteobacteria	17.3	0.65	17.4	0.27	26.1	1.94	39.5	2.42	56.2	1.03	88.2	1.85	27.8	1.93	86.9	0.11	63.1	5.90	46.9
Acidobacteria	29.6	1.35	38.3	2.66	24.5	1.80	23.0	1.64	7.19	0.16	1.25	0.09	1.24	0.35	0.73	0.02	5.28	0.01	14.6
Actinobacteria	7.51	0.35	2.58	0.03	1.68	0.09	2.65	0.12	1.72	0.06	1.45	0.15	54.0	8.26	3.41	0.29	6.14	0.33	9.01
Firmicutes	8.72	0.14	8.46	0.19	8.26	0.70	6.37	0.57	10.2	0.15	4.02	0.65	2.09	0.80	5.38	0.08	3.04	0.44	6.28
Bacteroidetes	1.30	0.12	1.72	0.43	1.36	0.36	3.77	0.57	7.27	1.14	3.36	0.60	0.83	0.49	0.30	0.01	13.7	3.11	3.73
Planctomycetes	7.40	0.15	4.16	0.11	3.15	0.43	2.05	0.19	0.35	0.02	0.09	0.01	0.16	0.01	0.07	0.01	0.67	0.30	2.01
Cyanobacteria	0.78	0.06	0.35	0.07	0.23	0.01	0.45	0.03	2.54	0.04	0.39	0.17	5.65	3.56	2.17	0.38	2.51	0.38	1.67

Bacteria genera	1 m	SE	2 m	SE	4 m	SE	6 m	SE	10 m	SE	20 m	SE	25 m	SE	40 m	SE	45 m	SE	Average
Acinetobacter	1.58	0.04	1.41	0.08	12.2	1.90	9.14	1.20	8.98	0.07	3.88	0.47	1.00	0.11	5.08	0.03	2.22	0.26	5.06
Acidovorax	0.36	0.03	0.21	0.01	0.58	0.04	1.32	0.32	0.99	0.03	12.6	1.52	0.33	0.01	3.82	0.04	6.73	1.45	3.00
Xenophilus	0.28	0.03	0.15	0.00	0.44	0.02	1.67	0.07	2.05	0.04	13.3	0.61	0.20	0.03	4.82	0.17	2.66	0.24	2.84
Novosphingobium	0.50	0.00	0.34	0.01	0.32	0.01	2.62	0.29	0.54	0.02	4.16	0.82	0.34	0.03	3.19	0.29	7.84	1.26	2.21
Pelomonas	0.11	0.01	0.05	0.00	0.15	0.01	0.30	0.04	0.62	0.01	17.6	0.48	0.12	0.01	0.18	0.01	0.60	0.11	2.19
Vulcaniibacterium	0.46	0.01	0.42	0.02	1.55	0.08	1.60	0.31	8.20	0.24	2.42	0.08	0.21	0.04	3.71	0.12	0.48	0.10	2.12
Anoxybacillus	0.95	0.04	1.08	0.10	1.68	0.13	2.24	0.32	6.84	0.05	2.67	0.32	0.47	0.08	0.97	0.01	0.65	0.10	1.95
Caulobacter	0.31	0.03	0.20	0.03	0.14	0.02	0.23	0.03	0.65	0.02	0.55	0.07	0.17	0.03	14.7	0.24	0.44	0.10	1.94
Alkanindiges	0.43	0.02	0.34	0.01	0.37	0.07	3.12	0.48	0.43	0.02	0.43	0.02	0.25	0.04	2.47	0.03	7.34	0.89	1.69
Cupriavidus	0.37	0.02	0.26	0.01	2.19	0.55	0.99	0.14	7.06	0.16	2.15	0.05	0.33	0.03	0.69	0.05	0.70	0.14	1.64
Gemmata	6.48	0.10	3.28	0.11	2.51	0.36	1.57	0.09	0.30	0.02	0.05	0.00	0.12	0.00	0.05	0.00	0.22	0.06	1.62
Phenylobacterium	0.21	0.01	0.11	0.01	0.13	0.01	0.22	0.01	0.60	0.00	0.28	0.01	0.16	0.04	11.8	0.27	0.38	0.06	1.54
Bradyrhizobium	0.37	0.01	0.13	0.01	0.14	0.02	0.89	0.14	0.33	0.00	3.60	0.56	0.63	0.42	4.25	0.10	2.68	0.90	1.45
Aquabacterium	0.18	0.01	0.12	0.00	0.39	0.05	0.83	0.11	2.69	0.12	3.08	0.27	0.19	0.04	2.60	0.05	1.96	0.22	1.34
Ralstonia	0.19	0.02	0.17	0.01	0.47	0.02	0.92	0.14	4.10	0.05	4.46	0.20	0.25	0.09	0.63	0.02	0.64	0.09	1.31
Pseudomonas	0.52	0.02	0.56	0.03	0.66	0.05	0.83	0.10	0.99	0.01	0.57	0.12	3.16	1.49	3.32	0.05	0.64	0.09	1.25
Undibacterium	0.25	0.02	0.21	0.01	0.28	0.02	2.10	0.29	2.80	0.11	1.20	0.03	0.18	0.02	0.14	0.01	3.95	0.70	1.23

Fungal phyla	1 m	SE	2 m	SE	4 m	SE	6 m	SE	10 m	SE	20 m	SE	25 m	SE	40 m	SE	45 m	SE	Average
Ascomycota	76.1	1.60	17.8	15.0	58.0	6.97	37.3	5.97	43.0	9.42	45.4	3.53	11.2	5.51	53.4	14.8	87.6	4.60	47.8
Basidiomycota	5.20	0.94	80.2	13.9	32.3	7.92	18.8	2.84	46.0	12.6	39.5	4.60	88.0	5.58	46.5	14.7	7.52	2.85	40.4
Fungal genera	1 m	SE	2 m	SE	4 m	SE	6 m	SE	10 m	SE	20 m	SE	25 m	SE	40 m	SE	45 m	SE	Average
Malassezia	2.87	0.50	55.5	18.1	22.2	6.76	9.01	0.90	18.6	6.49	25.1	9.74	65.7	5.25	45.1	15.6	2.22	0.67	27.4
Aspergillus	66.5	1.23	0.41	0.38	0.61	0.20	0.29	0.07	7.77	1.68	3.69	2.57	0.05	0.01	0.07	0.02	0.58	0.17	8.89
Coniosporium	1.67	0.20	0.06	0.02	1.79	0.49	4.63	0.33	0.77	0.06	9.09	3.68	3.51	3.38	7.82	7.71	40.0	7.10	7.70
Neurospora	1.62	0.20	0.01	0.01	1.45	1.30	0.11	0.05	0.14	0.01	0.62	0.32	0.02	0.00	0.02	0.01	28.6	4.46	3.62
Russula	0.17	0.03	0.02	0.01	0.25	0.09	0.30	0.09	6.04	5.78	0.19	0.03	11.9	5.95	0.03	0.02	0.21	0.06	2.12
Ophiostoma	0.05	0.00	0.00	0.00	0.03	0.01	0.03	0.01	13.5	6.67	0.04	0.01	0.00	0.00	0.01	0.01	0.62	0.14	1.59
Sporisorium	0.17	0.05	11.2	10.6	0.35	0.09	0.11	0.03	0.35	0.04	0.20	0.01	0.08	0.01	0.09	0.03	0.19	0.05	1.42
Peniophora	0.26	0.05	0.00	0.00	0.09	0.02	0.04	0.02	0.05	0.00	8.31	5.57	0.01	0.00	0.01	0.01	0.41	0.28	1.02

Only the dominant phyla/genera (average relative abundance > 1% across the profile) are shown, *SE: Standard error.

TABLE S5 Correlations between rare earth elements (REEs) and microbial community composition.

	Acidobacteria	*Firmicutes*	*Planctomycetes*	*Chloroflexi*	*Nitrospirae*	*Xenophilus*	*Gemmata*	*Massilia*
REEs	0.11	0.02	-0.04	-0.10	-0.04	-0.15	-0.05	-0.16
LREEs	0.26	0.18	0.11	0.06	0.10	-0.34	0.09	-0.02
HREEs	-0.32	-0.43	-0.43	-0.52	-0.41	0.45	-0.43	-0.48
$(LREEs/HREEs)_N$	0.81**	0.85**	0.86**	0.84**	0.76*	-0.67*	0.84**	0.74*
	Pseudarcicella	*Nitrospira*	*Curvibacter*	*Ktedonobacter*	*Rozellomycota*	*Trichoderma*	*Pyricularia*	*Microascus*
REEs	0.85**	-0.04	0.69*	-0.04	0.98**	0.82**	0.63	0.80**
LREEs	0.79*	0.10	0.62	0.10	0.92**	0.78*	0.49	0.73*
HREEs	0.74*	-0.41	0.66	-0.41	0.80*	0.66	0.80**	0.75*
$(LREEs/HREEs)_N$	-0.24	0.76*	-0.36	0.74*	-0.16	-0.22	-0.49	-0.18
	Strelitziana	*Tomentella*	*Phaeosphaeria*	*Pilatoporus*	*Entoloma*	*Lentinus*		
REEs	0.97**	0.93**	0.98**	0.98**	0.98**	0.98**		
LREEs	0.93**	0.89**	0.93**	0.93**	0.93**	0.92**		
HREEs	0.77*	0.74*	0.79*	0.78*	0.79*	0.80*		
$(LREEs/HREEs)_N$	-0.16	-0.18	-0.15	-0.12	-0.15	-0.16		

Only the phyla/genera with significant correlations to REEs are shown. The significance was checked by two-tailed test. *: $p < 0.05$; **: $p < 0.01$.

References

1. McCutcheon J, Southam G. 2018. Advanced biofilm staining techniques for TEM and SEM in geomicrobiology: Implications for visualizing EPS architecture, mineral nucleation, and microfossil generation. Chemical Geology 498:115-127.
